# From Water to Web: Trophic Transfer of Neonicotinoids from a Wastewater Effluent-Dominated Stream to Riparian Spiders

**DOI:** 10.1021/acsenvironau.5c00021

**Published:** 2025-07-22

**Authors:** Alyssa L. Mianecki, Jonathan R. Behrens, Dana W. Kolpin, Grant R. Hemphill, Krisha Kapoor, Gregory H. LeFevre

**Affiliations:** † Department of Civil & Environmental Engineering, 4083 University of Iowa, 4105 Seamans Center, Iowa City, Iowa 52242, United States; ‡ IIHR-Hydroscience &Engineering, 100 C. Maxwell Stanley Hydraulics Laboratory, Iowa City, Iowa 52242, United States; § University of Iowa Secondary Student Training Program, Belin-Blank Center, 600 Blank Honors Center, Iowa City, Iowa 52242, United States; ∥ Central Midwest Water Science Center, U.S. Geological Survey, 400 S. Clinton Street, Rm 269 Federal Building, Iowa City, Iowa 52240, United States; ⊥ Department of Biology, 3065 Duke University, 137 Biological Sciences Building, Durham, North Carolina 27708, United States

**Keywords:** riparian spiders, pharmaceuticals, neonicotinoids, wastewater, bioaccumulation, food webs, effluent-dominated streams

## Abstract

Municipal wastewater is a known point source of organic contaminants, including pharmaceuticals and neonicotinoid insecticides. Emergent aquatic insects can provide a direct aquatic-to-terrestrial contaminant transfer route to the food web, with implications for terrestrial food web dispersal of wastewater-derived organic contaminants. We quantified 17 target pharmaceuticals and insecticides (log *K*_ow_: −1.43 to 4.75) in surface water, fish, aquatic insects, and web-building riparian spiders at a wastewater effluent-dominated stream in eastern Iowa, USA. Two neonicotinoids, imidacloprid and clothianidin, had spider tissue concentrations of 8.9–84 ng/g and 1.2–11 ng/g, respectively. The imidacloprid/clothianidin ratios in spider tissues were reflective of the concentration ratios in the effluent-dominated streamwater and opposite of nearby agriculturally dominated waters. In contrast, no pharmaceuticals were detectable in the riparian spiders; however, only pharmaceuticals were present in both fish and aquatic insects (1.1–11 ng/g and 5.9–51 ng/g, respectively). Neonicotinoids are not predicted to enter aquatic food webs based on their log *K*_ow_ and bioconcentration factor values; therefore, an implication of this study is to warrant caution when using traditional bioaccumulation models for polar hydrophilic contaminants. This work provides further evidence that neonicotinoids undergo trophic transfer and represents the initial measurements, implicating such a transfer from effluent-dominated streams into terrestrial food webs. While this study emphasizes field-relevant observations, it is limited by environmental variability, including uncertainties in the biomass of emergent insects that likely contribute to spider diets. Future research could investigate contaminant metabolites within individual organisms or use complementary techniques to better understand the underlying mechanisms.

## Graphical abstract

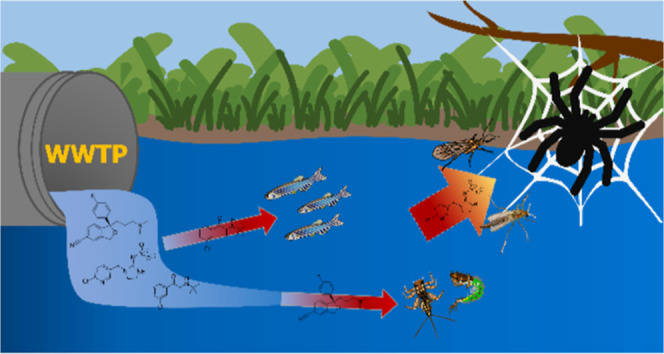

## Introduction

Wastewater effluent-dominated streams are an increasingly prominent human-impacted ecosystem^1^ and represent an overlooked exposure route of polar organic contaminants to aquatic and terrestrial organisms.^2–5^ Specifically, pharmaceuticals and urban-use pesticides, including neonicotinoid insecticides, are well-documented at high concentrations within effluent-dominated streams.^6–8^ These polar organic contaminants are often present as complex mixtures in surface waters, with the potential to generate adverse effects on aquatic and terrestrial biota. For example, pharmaceuticals can cause diminished predator avoidance behaviors, intersex features, reduced fecundity, and disrupted hormones in fish.^9–12^ Neonicotinoid insecticides can alter predator–prey interactions and reduce feeding and growth rates of aquatic insects.^13–16^ Additionally, these polar organic contaminants exhibit high mobility in the environment and are often characterized as pseudopersistent, where continuous inputs offset degradation rates (i.e., half-life: 2–6 days), resulting in relatively stable environmental concentrations.^2,17–19^ In effluent-dominated streams, sustained inputs from wastewater treatment plants maintain these concentrations, leading to prolonged biotic exposure well downstream of the point source.

Neonicotinoids are some of the most widely used insecticides in the world^20^ and have been detected in surface water,^7,21–23^ groundwater,^24^ wastewater,^25–28^ and drinking water.^29–31^ Neonicotinoid insecticides are highly polar, mobile, and negatively affect broader aquatic and terrestrial ecosystems.^7^ Two of the most commonly used neonicotinoids, imidacloprid and clothianidin, share an insecticidal mode of action^32^ and similar physical-chemical properties.^24^ The practical usage of these two common neonicotinoids is different; imidacloprid is often associated with urban land use from lawncare and indoor insect treatments, while clothianidin is more associated with agricultural land use from seed treatments in row-crop agriculture.^21,23,33^ We previously reported that municipal wastewater effluent can be a significant point source of imidacloprid to surface water.^6^ Nevertheless, broader ecological impacts of neonicotinoids originating from wastewater to riparian terrestrial ecosystems remain largely uninvestigated and may represent an unrecognized exposure pathway through the food web.

Biota living within or near effluent-dominated streams face consistent exposure to polar organic contaminants, which may accumulate in their tissues either through direct contact or via the food chain. Contaminant uptake by fish corresponds well with published bioaccumulation models that were developed for exposure routes involving direct contact with contaminants (i.e., ingestion, inhalation, and dermal exposure);^34,35^ however, these models do not accurately reflect processes relevant to emergent insects.^36^ Additionally, larval aquatic insects can directly accumulate pharmaceuticals in effluent-dominated streams and subsequently transfer contaminants to terrestrial environments as emerged flying adults.^37–41^ Emergent adult insects can subsequently spread contaminants to terrestrial organisms, including insectivorous riparian spiders.

Riparian spiders, notably those from the family Tetragnathidae (long-jawed orb weavers), are often abundant in many streamside habitats and are increasingly used as model organisms to monitor contaminant transfer from aquatic to terrestrial food webs.^42^ Riparian orb-weaving spiders are exposed to stream contaminants indirectly through their diet, which often consists of 50–100% of emergent insects, with higher reliance on emergent insects in more disturbed, urban streams.^43–45^ Larval aquatic insects can bioaccumulate polar organic contaminants through dermal absorption or dietary intake, and the extent of contaminant uptake may vary with feeding strategy—such as scraping, shredding, or predation—potentially influencing how these compounds are transferred throughout the food web.^38,46^ Riparian orb-weaving spiders can be exposed to contaminants originally present in the water when aquatic insects emerge and are caught in the riparian spider webs (located low above the water) and subsequently consumed.^47^ Increasingly, researchers are using tetragnathid spiders to quantify food web transfer of persistent bioavailable contaminants from surface waters.^47^ The presence of contaminants in riparian spiders may also point to a broader presence of contaminants throughout aquatic-terrestrial food webs. Measuring contaminants in riparian spiders to analyze food–web interactions has been applied to a diverse suite of contaminants, including mercury/other metals,^48,49^ PCBs,^50–52^ algal toxins,^53^ PFAS,^41,54^ pharmaceuticals,^38,55^ and agricultural pesticides.^56^ Although riparian spiders can be used to estimate contaminant trophic transfer between aquatic and terrestrial ecosystems, there is a dearth of spider studies examining pharmaceuticals,^38,55^ and none have investigated neonicotinoids from effluent-dominated streams. To fill this research gap, the overall objective of this work was to quantify both neonicotinoids and pharmaceuticals in tetragnathid spiders living adjacent to a temperate effluent-dominated stream with well-known municipal wastewater-derived neonicotinoid and pharmaceutical inputs.^5,6,57–63^ This is the first study to quantify neonicotinoid accumulation in riparian spiders living near an effluent-dominated stream, with pressing implications for the dispersal of wastewater-derived organic contaminants within terrestrial food webs.

## Materials and Methods

### Chemicals

Targeted chemicals were chosen for both water and tissue sample analysis based on historical occurrence and loading trends at the Muddy Creek study site in eastern Iowa, USA, from our prior research.^5,6,57–62,64^ The targeted chemicals for this study include 12 pharmaceuticals, 3 neonicotinoids, and 2 corrosion inhibitors. The full chemical information can be found in Table S2.

### Site Description and Sample Collection

Muddy Creek is an effluent-dominated stream located in the temperate ecoregion of east-central Iowa. Surface water grab samples were collected^5,6^ concurrently with biota in July 2019, August 2020, and July 2022 at four established U.S. Geological Survey (USGS) monitoring stations along the stream reach: (1) 100 m upstream of the wastewater treatment plant (WWTP) outfall (US1, 05454050), (2) at the WWTP outfall (EFF, 05454051), (3) 100 m downstream from the WWTP outfall (DS1, 05454052), and (4) 5.1 km downstream from the WWTP outfall (DS2, 05454090) (Section S1). Two sets of water samples were collected: one for pharmaceuticals that were analyzed by the USGS (Table S17),^65,66^ and another for pharmaceuticals and neonicotinoids analyzed by the University of Iowa (Table S16). Biota from Muddy Creek were collected from transects established around US1, DS1, and DS2 at the following times: tetragnathid spiders and emergent aquatic insects (adult stage) in August 2020, fish in July 2019, and aquatic insects (larval and adult stage) in July 2022 (Sections S3–S6). Muddy Creek is a diminutive, low-productivity stream with limited insect and spider biomass; the biomass collected here reflects the maximum effort expended each day of sampling.

In August 2020, tetragnathid spiders along the riparian zone of Muddy Creek were collected in 70–90 m transects established proximal to US1, DS1, and DS2 using methods adapted from Naslund et al.^49^ (Section S3). Spiders were collected with acid-washed bone tweezers starting at the stream surface up to 1 m horizontally and up to 2 m vertically from the water. The spiders were collected by hand (using tweezers, hands gloved), and each spider was stored in an individual 2 mL propylene vial and then placed on ice. A total of 434 tetragnathnid spiders were collected, yielding 2.24 g of dried biomass (Table S8). The collected spiders were pooled by sex^67^ and site into *n* = 11 composite samples (Table S9). Additionally, reference spiders were collected from a predominantly forested watershed with little agricultural activity in North Carolina (Section S3). Concurrently with spiders in August 2020, emergent insects were collected using floating emergence traps^68^ within the respective sampling transects (Table S12). A total of 287 emergent insects (representative of riparian spider diets^43–45^ ) were collected (252 Diptera, 28 Trichoptera, 6 Ephemeroptera, and 1 Odonata), yielding 0.25 g of dry biomass split into *n* = 3 composite samples (separated by site), which was insufficient for chemical analysis. Aquatic insects (collected in parallel timing with the sampling but not necessarily representative of riparian spider diets) were collected in July 2022 at US1, DS1, and DS2 using dip and kick nets. A total of 68 aquatic insects [32 Gerridae (surface dwelling nymphs/adults), 13 Odonata (swimming larvae), 7 Trichoptera (swimming larvae), and 16 from other orders] were collected from US1 and DS1, yielding 0.50 g of dry biomass in *n* = 4 composite samples (Tables S13 and S14). We functionally define “aquatic insects” as aquatic and semiaquatic insects that spend large portions of their life in the water and were in direct physical contact with the water at the time of collection. Fish were collected in July 2019 via an electrofishing survey at US1, DS1, and DS2 transects. A subset of composited fish samples (shiners, chubs, and minnows by site) was analyzed for a total of *n* = 26 samples, yielding 10.8 g of dry biomass (Table S15). Because the aquatic insects and fish spend substantial portions of their life cycle in the streamwater, the expected contaminant exposure is largely through physical contact (e.g., dermal absorption, gills)^35,43^ in contrast to riparian spiders, where no physical contact is expected and contaminant exposure would be through their diet of emergent insects.^44^ Thus, samples were collected with expectations of different exposure pathways.

### Analytical and Statistical Summary

Pharmaceutical water samples were sent to the USGS and analyzed using established USGS methods.^3^ Although we received results for all 210 compounds in the method (Table S17), only the 14 pharmaceuticals corresponding to the University of Iowa (UIowa) method^4^ were used to match the rest of the matrices. The UIowa pharmaceutical samples from August 2020 were not used because the samples became compromised due to the extended holding times during the COVID-19 pandemic. Neonicotinoid water samples were analyzed at UIowa as per previous research.^5^ Briefly, the 1 L samples were filtered (Whatman, GF/F), 50 ng of a deuterated surrogate standard mix was added for QC (quality control), and SPE (solid phase extraction) was performed using Waters HLB cartridges. After SPE, samples were evaporated to dryness and reconstituted in a 1 mL ACN/H_2_0 (1:1, v/v) solution. Spiders and other biota collected from Muddy Creek were extracted and analyzed using methods modified from LeFevre et al.^6^ Briefly, biota were lyophilized and homogenized with their same species/sex/site, and a sample (measured, 0.1–0.5 g) was extracted 3× with an acidified water/acetonitrile solution before filtering into the final sample vial. An isotopically labeled internal standard mix was added to all samples to check for matrix interferences prior to analyzing via LC–MS/MS. Biota extraction methods are fully detailed in Section S3. Chromatography was via Agilent 1290 HPLC with Eclipse Plus C18 column, and detection was via an Agilent 6460 Triple Quadrupole mass spectrometer. Specific details on ionization modes, chromatography methods, and instrument settings are in Table S4–S6. LC–MS/MS data analysis was conducted using an Agilent MassHunter Workstation Quantitative Analysis (version 10.1). All statistical analyses were performed in GraphPad Prism 9.0.0. Limits of detection in biota ranged from 4.8 ng/g to 23.6 ng/g, and limits of detection in water ranged from 0.48 ng/L to 2.36 ng/L (Table S7). Full analytical and statistical methods can be found in the Supporting Information (Section S2).

## Results and Discussion

### Neonicotinoids in Riparian Spiders and the Proximal Aquatic Environment

Imidacloprid and clothianidin were detected in tetragnathid spiders collected from Muddy Creek transects (Figure 1A), representing the first documentation of neonicotinoid occurrence in tetragnathid spiders living near a wastewater effluent-dominated ecosystem. Concentrations of imidacloprid in spider tissues ranged from 8.9 ng/g to 84 ng/g (*n* = 11/11 composite samples), and clothianidin ranged from <LOD-11 ng/g (*n* = 5/11 composite samples), similar in magnitude to tissue samples measured from a European agricultural stream system.^56^ The concentrations of neonicotinoids were not significantly different between male and female spiders at all sites combined (α = 0.05, two-tailed *p* = 0.8303, Table S9). No other organic contaminants analyzed (i.e., thiamethoxam, pharmaceuticals, and corrosion inhibitors) were detected in the spiders. Imidacloprid and clothianidin in spider tissues likely originate from their diet because the tetragnathid spiders inhabit webs near the water’s surface, where they capture emergent aquatic insects. In contrast, larval aquatic insects are exposed to contaminants through direct water contact (i.e., gills) and through their diets.^69–71^ Aquatic insects are known to bioaccumulate surface water contaminants, with some compounds biomagnifying across trophic levels to predators such as spiders.^37,42,72–74^

**Figure 1:**
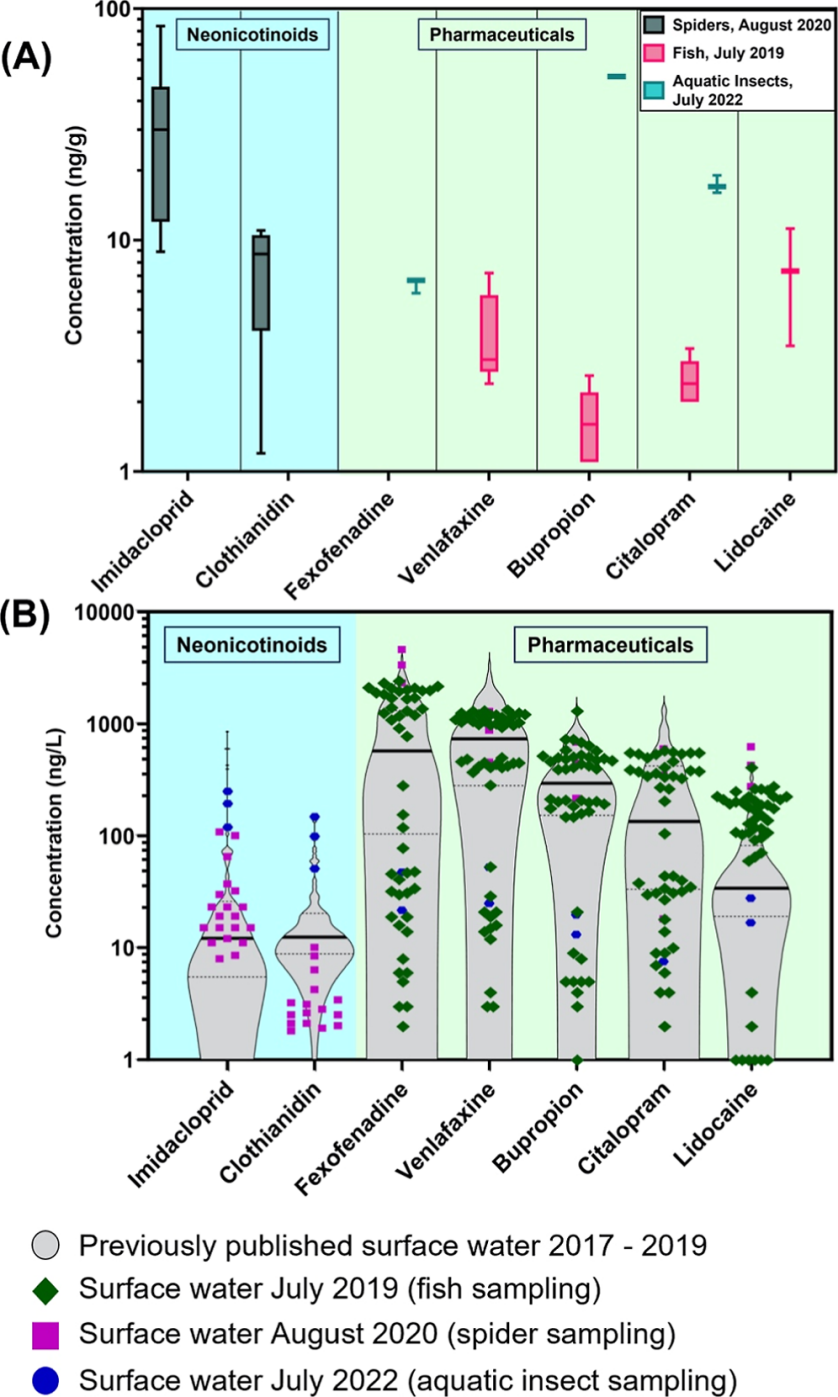
(A) Concentrations (ng/g) of select contaminants in biota tissues from Muddy Creek in eastern Iowa, USA. Error bars represent the minimum and maximum measured values. Boxes represent the interquartile range, central bars represent median values, and whiskers represent minimum and maximum values. Note log *y*-axis. Neonicotinoids (blue shaded) were only found within the spider tissues (black; 1.2 ng/g–84 ng/g). Pharmaceuticals (green shaded) were measured in both fish (pink; 1.1 ng/g–11 ng/g) and in aquatic insects (teal; 5.9 ng/g–51 ng/g; note, samples were inclusive of all aquatic insect samples collected [“aquatic insects” includes both aquatic and semiaquatic insects in physical contact with the water at the time of collection] and pooled). All other contaminants were not detected (Figure S6). Biota data separated by site transect are presented in the Supporting Information (Sections S3–S6)*.* (B) Contaminants in Muddy Creek are continuously released from the wastewater treatment plant effluent. Water samples from Muddy Creek were collected biweekly by University of Iowa (UIowa) researchers during 2017–2019 (gray shaded violin plots). Upper and lower quartiles are denoted by dashed lines and median values by a solid line. Water samples from July 2019 (green ◆) were collected by UIowa 3× per day for 6 days and coincide with collected fish samples. Water samples from August 2020 (purple ■) were collected once per day for 3 days by UIowa (neonicotinoids and corrosion inhibitors) and USGS (pharmaceuticals) and coincide with collected spider samples. Water samples from July 2022 (blue ●) were collected once on 1 day by UIowa and coincide with the collected aquatic insect samples. Water separated by site transect is presented in the Supporting Information (Section S7).

During our study, imidacloprid and clothianidin were ubiquitous in the streamwater, with imidacloprid concentrations averaging 18 ng/L across all sampling sites (7.9–37 ng/L) and clothianidin averaging 3.7 ng/L (1.8–10 ng/L; Table S16). These Muddy Creek water concentrations fall within our previously published ranges^6^ (imidacloprid 0.6–850 ng/L; clothianidin 3.4–134 ng/L; Figure 1B). Although somewhat speculative due to the small numbers of composite spider samples at each site, comparable ratios of imidacloprid/clothianidin (IMI/CLO) occurred in both the spiders and their respective surface water sites (Table 1). Neonicotinoids, including imidacloprid and clothianidin, share conserved toxicological modes of action (i.e., binding to the invertebrate nicotinic acetylcholine receptors) and have comparable physical–chemical properties. We would therefore expect in vivo IMI/CLO ratios to reflect ambient environmental conditions.^20^ At the most downstream site (DS2), imidacloprid was 4.6-fold higher than clothianidin in the surface water and 4.0-fold higher than in the spiders. At DS1, 100 m downstream from the WWTP, no clothianidin was detected within the spider tissues above the detection limit (4.8 ng/g; Tables S7 and S9), and the surface water IMI/CLO ratio was 7.0-fold. No spiders were collected directly from the effluent outfall (EFF); however, the IMI/CLO ratio was the highest in the water at 9.4-fold. Finally, at US1 (upstream from the WWTP), the IMI/CLO ratio in the spiders was 9.6-fold; however, the water ratio was only 1.4-fold. US1 and EFF are 100 m apart along the stream reach but only 55 m apart overland. We therefore speculate that a portion of the spiders’ diets is subsidized by emergent insects that spent portions of their larval lifecycles in the water impacted by effluent, and dispersal of 1–2 km is possible (i.e., drift paradox).^75,76^ Indeed, the surface water IMI/CLO ratios of other agricultural watersheds around Iowa are inverted compared to the effluent-dominated watershed at Muddy Creek: clothianidin is more frequently detected and at higher concentrations than imidacloprid.^6,77^ Specifically, USGS studies have reported^22,29^ an average IMI/CLO ratio of 0.5-fold in Iowa agricultural watersheds, including in the nearby Iowa River, compared to the average 5.0-fold ratio we measured in Muddy Creek. If the spiders were exposed to neonicotinoids outside of their diet or if their diets were subsidized by terrestrial insects from the broader watershed, we would have expected to find consistently higher levels of clothianidin because of the influence of local agriculture. In contrast, we consistently observed higher levels of imidacloprid in the spiders, indicating that the riparian spiders are likely being exposed to neonicotinoids originating from WWTP effluent discharged into Muddy Creek rather than from upstream agricultural inputs.

**Table 1:**
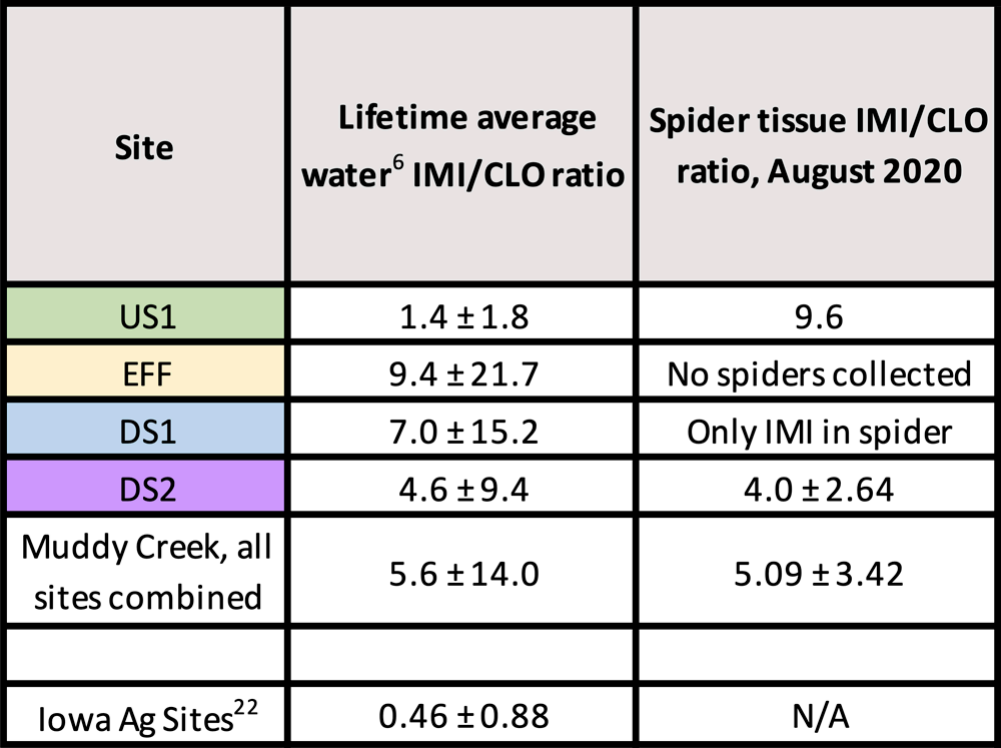
Lifetime Ratios of Imidacloprid/Clothianidin (IMI/CLO) in Water Compared to the IMI/CLO Ratios Found in Spiders in Muddy Creek^a^

a Also, see Table S10 for more comprehensive results.

We previously demonstrated^6^ that the municipal effluent is a significant point source mass load of imidacloprid to the Muddy Creek study site and that comparatively lower clothianidin loads are largely associated with upstream sources. The consistent IMI/CLO chemical ratios between surface water and spider tissue concentration provide indirect evidence that the neonicotinoids undergo trophic transfer from the effluent-dominated surface water through the food chain into the riparian spiders. We acknowledge that the low biomass of emergent flying insects that could be collected during the sampling time, which likely comprise the riparian spider diets, limits our ability to directly trace the trophic transfer from water to insects to spiders. Despite the low biomass of emergent insects that could be collected (potentially due to elevated levels of neonicotinoids, as described below), we do not believe that the spider diets reflected the substantial influence of terrestrial insects in their diets. As described above, riparian orb-weaving spiders’ diets often consist of 50–100% of emergent insects, with a reported higher reliance on emergent insects in more disturbed, urban streams.^40–42^ Furthermore, the neonicotinoid IMI/CLO ratio in the spider tissues reflects the neonicotinoid ratios in the effluent-dominated stream rather than the broader watersheds. Therefore, we believe that the low biomass of emergent insects collected during the sampling period likely reflects lower insect populations, possibly due to chemical exposure or habitat limitations, as described elsewhere. This may lead to lower riparian spider populations compared to other streams, but it likely does not indicate a change in the riparian spider diets. It is possible that some Gerridae fed on aquatic insects and that their exposure may have resulted from diets more like those of riparian spiders than those of aquatic insects; however, consideration of instar stage or ontogenetic dietary shifts in macroinvertebrates was beyond the scope of this study. Future studies may be considered using stable isotope probing, high-resolution mass spectrometry for metabolites, insect life stages, or bench-scale laboratory work to supplement the in-field findings.

### Pharmaceuticals in Aquatic Biota and in the Surface Water

We previously established that the WWTP effluent is a consistent point source of pharmaceuticals to Muddy Creek water.^5,64^ In Muddy Creek, there is a strong pharmaceutical concentration gradient with negligible exposure upstream from the WWTP outfall (US1), and then a large increase in exposures downstream from the outfall (DS1), followed by general attenuation with further downstream transport (DS2). The observed pattern was maintained for all sampling events pre- and post-COVID-19 pandemic. The concentration of pharmaceuticals monitored in this study was not expected to change due to the COVID-19 pandemic, likely due to the types of pharmaceuticals monitored.^78,79^ The subsequent water sampling events collected concurrently with the spider, fish, and aquatic insect samples were consistent with our prior reported^5,64^ pharmaceutical patterns and abundance (Figure 1B). Other researchers have reported pharmaceuticals in riparian spider tissues;^38,80^ we had therefore hypothesized that pharmaceuticals would be present in riparian spiders near Muddy Creek due to the high pharmaceutical loads present in the stream reach (total pharmaceutical concentrations ranged from 7010 to 49,800 ng/L;^5^ maximum concentrations of the neonicotinoids imidacloprid and clothianidin were 116 ng/L and 92.8 ng/L, respectively). Surprisingly, no target pharmaceuticals were detected in the spiders. Unexpectedly, we detected sulfamethoxazole in the female reference spiders from North Carolina (*n* = 2/2, 45.5 ng/g) even though the site is predominantly forested with some rural septic-based communities; sulfamethoxazole is a very common human- and animal-use antibiotic (Section S3). In contrast to the spiders, our limited samples of aquatic insects and fish from Muddy Creek yielded traces of pharmaceuticals (Figure 1A). Three pharmaceuticals were detected in composited aquatic insect samples (Table S14 mass weighted average; bupropion 17 ng/g, *n* = 1/4; citalopram 17 ng/g, *n* = 3/4; fexofenadine 6.4 ng/g, *n* = 3/4; top families including Gerridae, Odonata, Trichoptera), and four pharmaceuticals were detected in composited fish samples (Table S15 mass weighted average; bupropion 1.7 ng/g, *n* = 15/26; citalopram 2.6 ng/g, *n* = 7/26; lidocaine 11 ng/g, *n* = 2/26; venlafaxine 4.1 ng/g, *n* = 14/26; shiners, chubs, minnows). Although these tissue samples were collected at separate times, our prior work demonstrated that pharmaceutical levels in Muddy Creek exhibit year-over consistency^5,62^ (Figure 1B). Because we have multiple years of water quality monitoring data available at the site (Figure S6), we expect the tissue samples collected to represent comparable biological uptake for neonicotinoids and pharmaceuticals at the same effluent-dominated stream.

### Contaminant Mixture Representation at Different Trophic Levels

Muddy Creek exhibited varying contaminant compositions, and some chemicals in the water were not detected in the food web. Previously published work^5,6^ demonstrates that Muddy Creek contains complex mixtures of polar organic contaminants including pharmaceuticals, neonicotinoids, and corrosion inhibitors. The mixture representation of these contaminants in Muddy Creek surface water (based on the proportion of total concentration) downstream from the outfall is approximately 94% pharmaceuticals, 5% corrosion inhibitors, and 1% neonicotinoids. This mixture, however, was not similarly reflected in the biota tissues; the contaminants measured within the fully aquatic biota (i.e., aquatic insects, fish) comprised a 100% pharmaceutical mixture, whereas the riparian spiders comprised a 100% neonicotinoid mixture (Figure 2A). Comparatively, the contaminants (the sum of all three categories of chemicals measured) in surface water (Figure 2B) are on average 5.2 ng/mL (ppb) throughout our study period. The pharmaceuticals found in the fish were detected at an average of 7.8 ng/g (ppb) and in the aquatic insects at an average of 34.3 ng/g (ppb). Neonicotinoids were detected in the spiders at an average of 40.2 ng/g (ppb). The average contaminant concentration was consistently higher than that found in the surface water, on a part-per-billion basis. These results indicate distinct differences in the types and levels of contaminants present in biota tissues.

**Figure 2:**
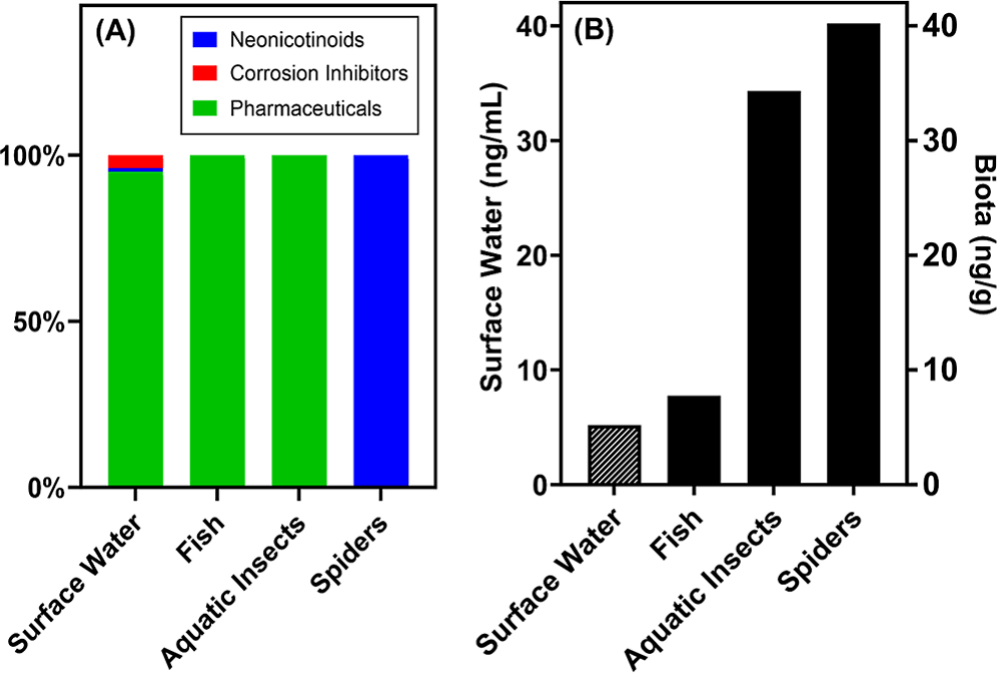
(A) Proportional composition of contaminants differed from surface water compared to each biological matrix. The surface water is dominated by 94% pharmaceuticals (green), 5% corrosion inhibitors (red), and 1% neonicotinoids (blue); aquatic insects contained 100% pharmaceuticals and riparian spiders contained 100% neonicotinoids. (B) Comparatively, the contaminants (the sum of all three categories of chemicals measured) in the surface water (hatched symbol bar, left *Y* axis, average values) are on average 5.2 ng/mL (ppb) throughout our study period. The pharmaceuticals found in the fish (solid, right *Y* axis, average values) were detected at an average of 7.8 ng/g (ppb) and in the aquatic insects at an average of 34.3 ng/g (ppb; note, samples were inclusive of all aquatic insect samples collected). Neonicotinoids were detected in the spiders at an average 40.2 ng/g (ppb). The average contaminant concentration was consistently higher than what is found in the surface water, on a part-per-billion basis.

Species-specific variations in contaminant uptake/concentration in larval aquatic insects and fluctuations in contaminant levels from larvae to emergent adults during metamorphosis may influence the differences between aquatic and terrestrial contaminant mixtures.^81–83^ Studies have also found that some pharmaceuticals may undergo trophic dilution, where concentrations decrease up the food chain.^38^ Additionally, the majority of collected aquatic insects belonged to the orders Gerridae, Odonata, and Trichoptera; however, emergent insects that better represent the diets of tetragnathid spiders^45^ were likely predominantly Dipterans. It is highly unlikely that tetragnathids predate on Gerridae (water-strider) or Odonata (dragonfly) due to their sizes; tetragnathids may predate on some smaller Trichopterans (caddisflies), but most of the biomass tetragnathids consuming in Muddy Creek is presumed from Diptera (small midge flies). Despite extensive sampling efforts, no larval Dipterans were collected from Muddy Creek, and the >200 adult Dipterans collected did not yield sufficient biomass to detect any targeted contaminants above the limits of detection. Other studies with abundant Dipterans have indicated that these insects can be substantial exporters of pesticides from surface water.^84^ Adult emergent insects were composited on a site-by-site basis and yielded only 0.02 g to 0.03 g of dry mass per site (Table S12). Our extraction method was optimized for a dry mass of 0.2 g and provided limits of detection in the single nanogram per gram range (Table S7). Muddy Creek is a small and relatively unproductive stream; throughout our sampling, we used exhaustive efforts to collect nearly all visible insects within a transect on a single day (Figure S2 for visuals). Additionally, our past studies in Muddy Creek have determined that the WWTP occasionally discharges neonicotinoids at concentrations higher than chronic US EPA aquatic life benchmarks for aquatic invertebrates (10 ng/L for imidacloprid and 50 ng/L for clothianidin) and sometimes even above acute benchmarks (385 ng/L for imidacloprid).^6^ This means that there could have been insect die-offs that have gone undetected directly during this study, potentially changing the represented insect population in the effluent-dominated stream compared with unaffected streams. The diet of the fish collected could not be determined conclusively from this study; many small fish consume insects of various life stages, but fish can also be exposed to contaminants through direct physical contact. The representation of contaminant mixtures at different trophic levels within Muddy Creek emphasizes the complexities of contaminant transfer from aquatic ecosystems.

Neonicotinoids, corrosion inhibitors, and most pharmaceuticals are relatively polar compounds (log *K*_ow_ values range^85^ of compounds measured: −0.9 to 4.5) with low predicted likelihoods of bioaccumulation (Figure 3). Yet, we measured both neonicotinoids and pharmaceuticals in biota from Muddy Creek, demonstrating that traditional bioaccumulation models may be limited for more polar contaminants—particularly for neonicotinoids. Traditional contaminant bioaccumulation models (e.g., pp-LFER/LSERs) work well for aquatic organisms, like fish, because relationships built on well-studied values (hydrophobicity, pH, log *K*_ow_) can be used.^35^ These models were originally developed for legacy persistent organic pollutants, rather than pseudopersistent polar organic contaminants.^17,18,86^ Traditional models are less accurate, however, when transitioning from aquatic to terrestrial ecosystems because many of the underlying assumptions are different (i.e., biota are not in constant contact with the contaminated media or contaminants may be selectively excreted during metamorphosis).^83,87^ Laboratory and field studies, such as this investigation, are more representative of the real potential for an individual contaminant to bioaccumulate and move throughout the environment and are thus critical for actual exposure assessment. For example, imidacloprid has a reported log *K*_OW_ of 0.57 and a predicted^19,85^ BCF of 6.44 L/kg (Figure 3). These predicted BCF values indicate that imidacloprid should be well within the “low risk” category (high risk is defined^88,89^ as BCF ≥ 1000 or log *K*_OW_ ≥ 4.2); however, imidacloprid has been repeatedly demonstrated to bioaccumulate in aquatic ecosystems, including in Muddy Creek.^56,70,90,91^ This phenomenon may be due to a novel accumulation mechanism recently proposed by Raths et al.,^92^ where thiacloprid (a structurally similar neonicotinoid) is irreversibly bound to invertebrate nicotinic acetylcholine receptors, preventing toxin elimination. Additional studies demonstrate that thiacloprid exhibits elimination resistance in aquatic invertebrates compared to other pesticides.^93^ Imidacloprid and clothianidin share a common mode of action and similar physicochemical properties (e.g., solubility, *K*_ow_)^20^ with thiacloprid, suggesting that their presence in riparian spider tissues may result from limited elimination, as has been reported for thiacloprid.^92^ This phenomenon may drive the high levels of neonicotinoids that we observed at the effluent-dominated stream compared to pharmaceuticals. Analogously, neonicotinoids are not predicted to sorb extensively due to their low log *K*_ow_ values, but neonicotinoids do successfully sorb to activated carbon via electrostatic mechanisms that are often unaccounted for in traditional sorption models.^30^ Therefore, future predictive studies for emerging contaminants that hold target-specific binding mechanisms such as neonicotinoids may benefit more from QSAR-based models^94^ rather than pp-LFER/LSER-based approaches.

**Figure 3:**
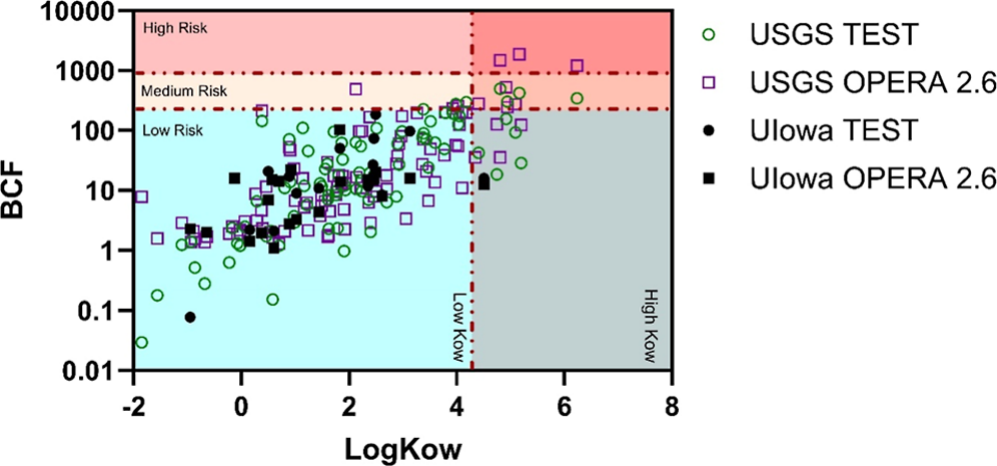
Log *K*_ow_ and bioaccumulation factors (BCFs) are commonly applied reference values to predict the accumulation of a substance into an organism. Here, the USGS method^65^ list and UIowa method^5^ list compound predicted values are plotted. Using EPA CompTox Chemical Dashboard^19,85^ to search databases, log *K*_ow_ and BCF values from OPERA 2.6 ^95^ (open source QSAR model) and TEST^96^ (Toxicity Estimation Software Tool) were sourced and plotted. In this plot, USGS refers to the suite of compounds measured using the USGS pharmaceutical method^65^ and UIowa refers to the 17 compounds analyzed in-house at the University of Iowa.^5,6^ When BCF and log *K*_ow_ are plotted against each other, areas of low vs high accumulation potential can be extrapolated. For log *K*_ow_, values less than 4.2 are considered low risk.^88,89^ For BCF, values less than 250 are considered low risk and values over 1000 are high risk.^88,89^ From these extrapolations of the UIowa list of contaminants, only fexofenadine falls into high risk based on log *K*_ow_, and no contaminants exceed low risk from a BCF standpoint. However, multiple neonicotinoids and pharmaceuticals were measured in biota from Muddy Creek and have been reported in biota elsewhere.^38,55,56^

## Conclusions

To our knowledge, this research is the first known report of neonicotinoid trophic transfer from an effluent-dominated stream to riparian orb-weaving spiders. Neonicotinoid transfer from effluent-dominated streams to riparian orb-weaving spiders represents one of many potential pathways of exposure of neonicotinoid to the broader ecosystem. The presence of neonicotinoids in riparian orb-weaving spiders suggests a relatively rapid transfer of the species from surface water through the food chain. This inference is supported by the spiders’ life cycle in which eggs are laid in the fall, adults die off thereafter, and the young hatch in the spring. Contaminant accumulation in riparian spiders may also imply that other insectivorous terrestrial animals are exposed to effluent-derived contaminants. Animals including amphibians, birds, and bats are documented to bioaccumulate effluent-derived contaminants, demonstrating the broad reach of effluent-derived chemical exposures in the environment.^41,52,70,74,97^ The movement of contaminants up the aquatic-terrestrial food web indicates that effluent-dominated streams could represent a diffuse source of contaminants to wildlife, livestock, and humans along their reach.

We previously demonstrated that differential attenuation of compounds within the complex mixture drives spatiotemporal evolution of exposure conditions along the Muddy Creek study reach,^5,58^ and here, we reveal that changing complex mixture chemical composition representation may also occur when moving through the aquatic-terrestrial trophic food chain. Effluent-dominated streams generate unique exposure conditions for aquatic organisms due to the pseudopersistent contaminant exposure conditions.^17,18,86^ Compared to nearby agriculturally dominated systems (maize and soybean), where pesticide contamination is largely driven by small runoff events,^98^ effluent-dominated streams can receive relatively consistent daily chemical inputs of pesticides.^6,22,99^ Biota exposed to organic contaminants in effluent-dominated streams face both a higher baseline chronic exposure risk over time and an additional acute risk during pulse events in the watershed. The transfer of pharmaceuticals from water to aquatic organisms (i.e., fish, insects) has been well-established and generally agrees with conventional bioaccumulation prediction models.^42,55,100^ Still, emerging research demonstrates that neonicotinoid bioaccumulation or trophic transfer cannot be effectively predicted using conventional approaches.^56,92,93,101^ The presence of neonicotinoids rather than pharmaceuticals in spider tissues may reflect novel accumulation mechanisms recently reported in the literature, as described above. Although this study was focused on the important first measurements associated with field relevance and has limitations associated with the natural environment, future work could examine metabolites of contaminants (i.e., high resolution mass spectrometry) within individual organisms or employ additional techniques (i.e., stable isotope tracing, bench-scale uptake testing, and trophic transfer studies/modeling) to further illuminate mechanisms. The findings from this study highlight previously overlooked contaminant pathways to the aquatic ecosystem and food web and emphasize the importance of moving beyond traditional predictive models, particularly in understanding the exposure risks to broader terrestrial ecosystems impacted by wastewater effluent.

## Supplementary Material



## Notes

Additional site descriptions, chemical and instrument analysis parameters and details, biota and water collection and extraction procedures and results, environmental modeling description, and results (PDF)
