# Supplementary material for: From Water to Web: Trophic Transfer of Neonicotinoids from a Wastewater Effluent-Dominated Stream to Riparian Spiders
Source: ACS Environ Au. 2025 Jul 22;5(5):457–67. doi: 10.1021/acsenvironau.5c00021 (PMC12447229; doi:10.1021/acsenvironau.5c00021)
Supplement: Supplementary file 1 [file vg5c00021_si_001.pdf]

Supporting Information

From Water to Web: Trophic Transfer of Neonicotinoids from a Wastewater Effluent-Dominated Stream to Riparian Spiders

Alyssa L. Mianecki[§¶], Jonathan R. Behrens[#], Dana W. Kolpin[Δ], Grant R. Hemphill[§],

Krisha Kapoor[¥], Gregory H. LeFevre[§¶*]

[§]Department of Civil & Environmental Engineering, University of Iowa, 4105 Seamans Center, Iowa City, IA 52242, United States; [¶]IIHR-Hydroscience &Engineering, 100 C. Maxwell Stanley Hydraulics Laboratory, Iowa City, IA 52242, United States; [Δ]U.S. Geological Survey, Central Midwest Water Science Center, 400 S. Clinton St, Rm 269 Federal Building, Iowa City, IA 52240, United States; [#]Department of Biology, Duke University, 137 Biological Sciences Building Durham, NC 27708, United States; [¥]University of Iowa Secondary Student Training Program, Belin-Blank Center, 600 Blank Honors Center, Iowa City, Iowa 52242, United States

***Corresponding Author:** GHL: gregory-lefevre@uiowa.edu; Phone: 319-335-5655; 4105 Seamans Center for Engineering, University of Iowa, Iowa City IA, United States

Any use of trade, firm, or product names is for descriptive purposes only and does not imply endorsement by the U.S. Government or the authors.

Includes: 34 total pages (inclusive of this page), 6 figures, and 18 tables.

Table of Contents

SECTION S1: SITE DESCRIPTION 2

SECTION S2: CHEMICALS & INSTRUMENT PARAMETERS 5

SECTION S3: SPIDER Collection, Extraction, Results 8

SECTION S4: EMERGENT MACROINVERTEBRATE Collection, Extraction, Results... 14

SECTION S5: AQUATIC MACROINVERTEBRATE Collection, Extraction, Results 16

SECTION S6: FISH Collection, Extraction, Results 18

SECTION S7: SURFACE WATER Collection, Extraction, Results 21

SECTION S8: REFERENCES 32

SECTION S1: SITE DESCRIPTION

Study Site Description. Muddy Creek (North Liberty and Coralville, Iowa, USA; (Latitude 41°42'00", Longitude 91°33'46") has a drainage area of 22.5 km^2 and is impacted by both agricultural and urban land use (**Figure S1, Table S1**). Muddy Creek is substantially impacted by treated wastewater from the North Liberty Wastewater Treatment Plant (WWTP). The WWTP was built in 2008 and uses a modern membrane reactor to remove particles >0.004 µm in addition to biological nitrogen and phosphorus removal for the municipality of 21,399.[1] Streamflow is monitored at U.S. Geological Survey (USGS) gaging station 05454090.[2]

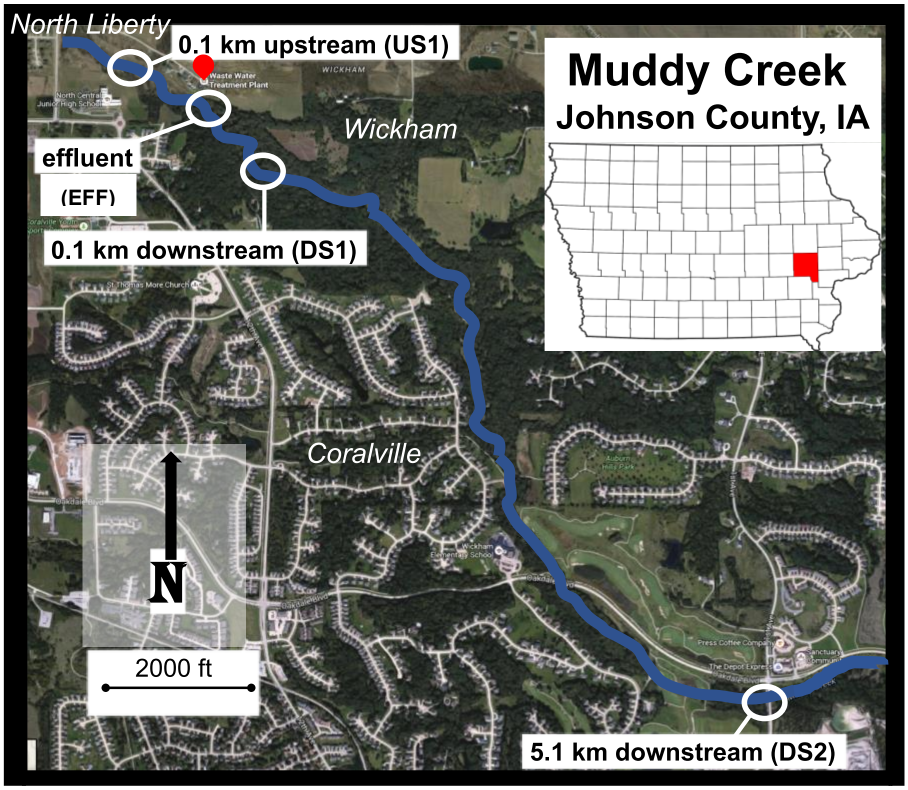

Figure S1 Map of Muddy Creek in the cities of North Liberty and Coralville, Iowa. The red circle indicates the location of the North Liberty WWTP. Water samples were collected at US1 (0.1 km upstream of the WWTP outfall, 05454050), EFF (effluent outfall, 05454051), DS1 (0.1 km downstream from the effluent outfall, 05454052), and DS2 (5 km downstream from the effluent outfall, USGS gaging station, 05454090). Biota sampling transects of approximately 100 m were established surrounding US1, DS1, and DS2.

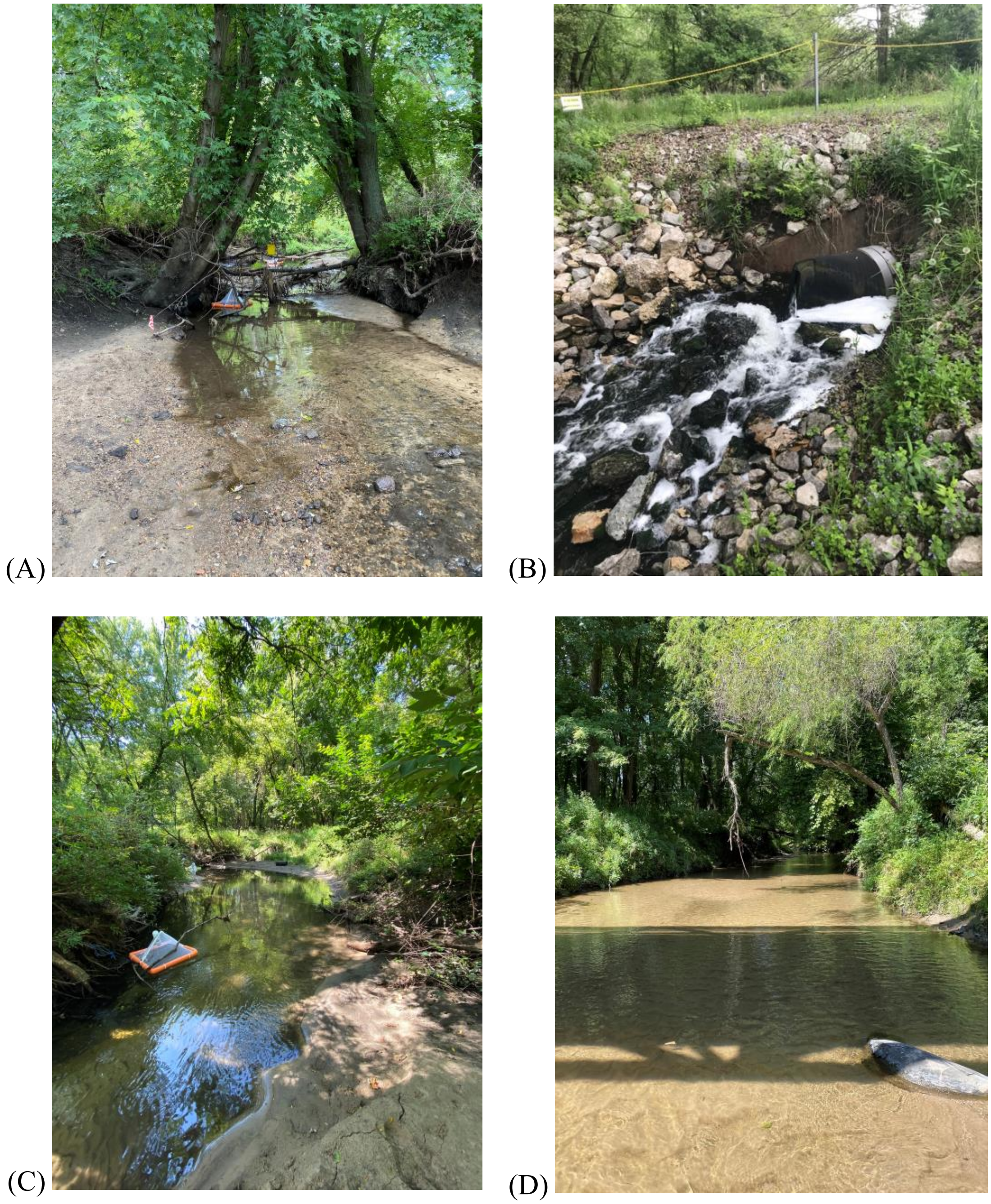

Figure S2 Photos of the sampling sites (A) 0.1 km upstream (US1, 05454050), (B) the effluent outfall (EFF, 05454051), (C) 0.1 km downstream (DS1, 05454052), and 5.1 km downstream (DS2, 05454090). Photos were taken by the authors on August 6th, 2020.

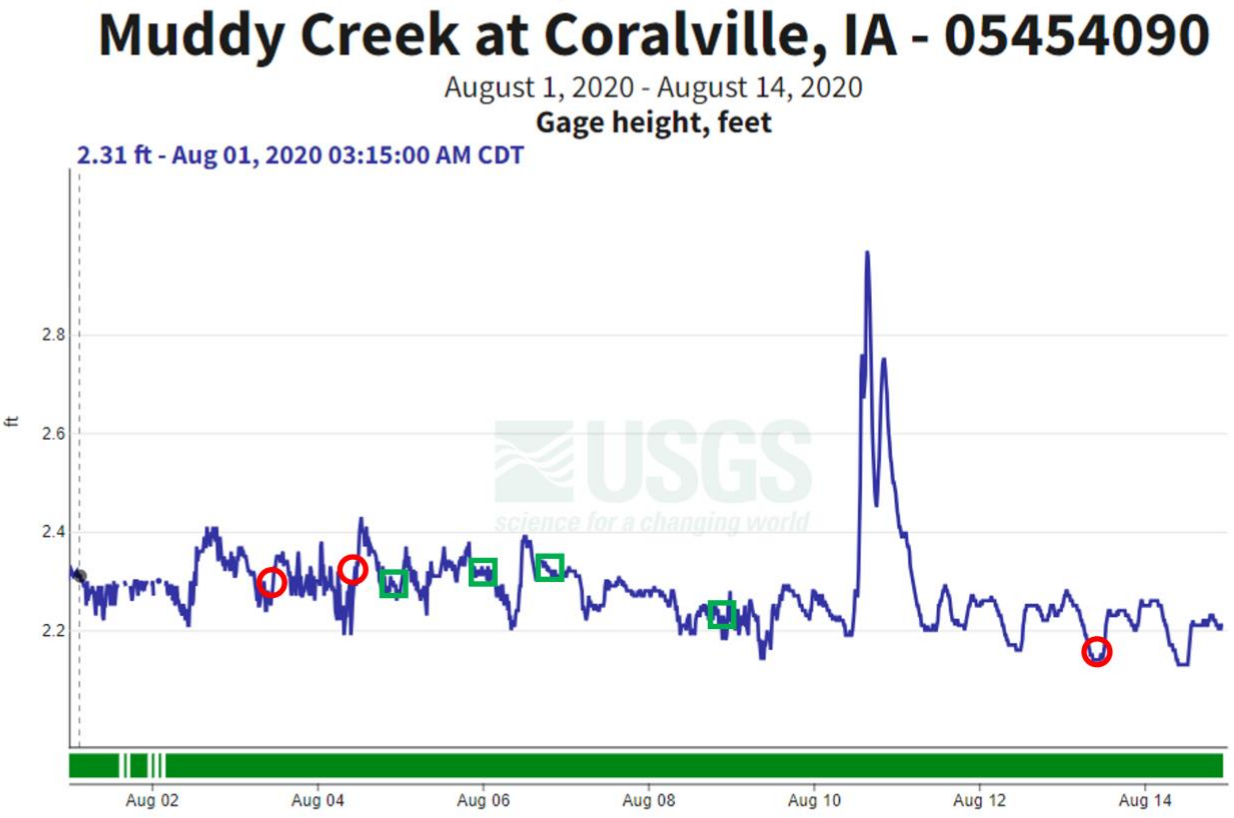

Figure S3 Gage height measured at DS2 in Muddy Creek (gage height, ft)[2] during the spider sampling. Water samples were collected 3 times in the morning, as denoted by the red circles. Spider samples were collected 4 times in the evening after dark, as denoted by the green squares. Streamflow was measured manually at US1 and DS1 on August 6th, 2020 between 2 and 3 pm. At US1, the flow was 1.807 ft^3/s and at DS1 the flow was 4.532 ft^3/s, translating to roughly 40% effluent in the stream downstream from the outfall during that time point.

Table S1 Land use information for Muddy Creek, determined using USGS National Land Cover Database. (https://www.usgs.gov/centers/eros/science/national-land-cover-database?qt-science_center_objects=0#qt-science_center_objects)

Station Name	Open Water	Developed	Barren Land (Rock/Sand/Clay)	Forest	Shrub/ Scrub	Grassland/ Herbaceous	Pasture/ Hay	Cultivated Crops	Wetlands
US1 (05454050)	0.13%	72.49%	0.07%	1.69%	0.00%	1.61%	2.82%	20.72%	0.44%
Effluent (05454051)	0.13%	72.50%	0.07%	1.70%	0.00%	1.61%	2.81%	20.69%	0.46%
DS1 (05454052)	0.13%	72.32%	0.07%	2.00%	0.00%	1.60%	2.86%	20.52%	0.47%
DS2 (05454090)	0.40%	59.97%	0.04%	12.36%	0.02%	2.38%	7.06%	17.45%	0.31%

SECTION S2: CHEMICALS & INSTRUMENT PARAMETERS

Table S2 Target compounds analyzed in the biota and in the water of Muddy Creek are listed below. These are the same chemicals as analyzed during prior studies at Muddy Creek.[4,5]

Compound	Category	CAS No.	Manufacturer
Bupropion	Pharmaceutical	31677-93-7	Sigma-Aldrich
Carbamazepine	Pharmaceutical	298-46-4	Sigma-Aldrich
Citalopram hydrobromide	Pharmaceutical	59729-32-7	Sigma-Aldrich
O-desvenlafaxine hydrochloride	Pharmaceutical	300827-87-6	Sigma-Aldrich
Fexofenadine hydrochloride	Pharmaceutical	153439-40-8	Sigma-Aldrich
Fluconazole	Pharmaceutical	86386-73-4	Sigma-Aldrich
Guanlyurea	Pharmaceutical	141-83-3	Sigma-Aldrich
Lidocaine	Pharmaceutical	137-58-6	Sigma-Aldrich
Metformin hydrochloride	Pharmaceutical	1115-70-4	Sigma-Aldrich
Methocarbamol	Pharmaceutical	532-03-6	Sigma-Aldrich
Sulfamethoxazole	Pharmaceutical	723-46-6	Sigma-Aldrich
Venlafaxine hydrochloride	Pharmaceutical	99300-78-4	Sigma-Aldrich
1H-Benzotriazole	Corrosion Inhibitor	95-14-7	Sigma-Aldrich
5-Methyl-1H-Benzotriazole	Corrosion Inhibitor	136-85-6	Sigma-Aldrich
Thiamethoxam	Neonicotinoid Insecticide	153719-23-4	Sigma-Aldrich
Imidacloprid	Neonicotinoid Insecticide	138261-41-3	Sigma-Aldrich
Clothianidin	Neonicotinoid Insecticide	210880-92-5	Sigma-Aldrich
Metformin-d_6	QC Standard	1185166-01-1	Sigma-Aldrich
Bupropion-d_9	QC Standard	1189725-26-5	Sigma-Aldrich
Carbamazepine-d_{10}	QC Standard	132183-78-9	Sigma-Aldrich
Citalopram-d_6	QC Standard	1190003-26-9	Sigma-Aldrich
Venlafaxine-d_6 hydrochloride	QC Standard	1062606-12-5	Sigma-Aldrich
Imidacloprid-d_4	QC Standard	1015855-75-0	Sigma-Aldrich
Thiamethoxam-d_3	QC Standard	1294048-82-0	Sigma-Aldrich
Caffeine^{13}-C_3	QC Standard	78072-66-9	Sigma-Aldrich

Table S3 Solvents used for extraction and on LC/MS/MS for analysis.

Solvent	Grade	Manufacturer
Acetonitrile	Optima LC/MS	Fisher, Fair Lawn, NJ
Formic Acid	Optima LC/MS	Fisher, Fair Lawn, NJ
Methanol	Optima LC/MS	Fisher, Fair Lawn, NJ
Water	Optima LC/MS	Fisher, Fair Lawn, NJ

Table S4 Agilent 1290 HPLC flow gradient settings are provided below. The gradient was adapted from the USGS pesticide method 2437[7] and paired with an Eclipse Plus C18 Column.

Time	A (Water + 0.1% formic Acid)	B (ACN)	Flow
4.00 min	80.0%	20.0%	0.400 mL/min
5.00 min	60.0%	40.0%	0.600 mL/min
10.00 min	40.0%	60.0%	0.600 mL/min
15.00 min	20.0%	80.0%	0.600 mL/min
16.00 min	10.0%	90.0%	0.600 mL/min
16.10 min	90.0%	10.0%	0.600 mL/min

Table S5 Agilent 6460 Triple Quadrupole mass spectrometer instrumental setting are provided below. These settings were used for all LC-MS/MS analyses.

MS/MS Parameters	
Gas Temp (°C)	300°C
Gas Flow (L/min)	5 L/min
Nebulizer Pressure	45 psi
Sheath Gas Temp	250 °C
Sheath Gas Flow	11 L/min
Capillary Voltage (+/-)	3500/3500 V
Nozzle Voltage (+/-)	500/500 V

Table S6 Multiple reaction monitoring mode (MRM) transitions for the Agilent 6460 Triple Quadrupole system. Each chemical is broken into two product ions which are listed below.

Compound Name	Time segment	Precursor Ion (m/z)	Product Ion (m/z)	Dwell (ms)	Fragmentation energy (V)	Collision Energy (V)	Cell Accelerator (V)	Polarity
Metformin-d6	2	136.15	77.1	75	63	25	4	Positive
Metformin-d6	2	136.15	60.1	75	63	12	4	Positive
Metformin	2	130.11	71.1	75	65	24	4	Positive
Metformin	2	130.11	60.1	75	65	12	4	Positive
Guanlyurea	2	103.06	60.1	75	65	8	4	Positive
Guanlyurea	2	103.06	43.1	75	65	25	4	Positive
Fexofenadine	3	502.3	466.3	40	146	28	4	Positive
Fexofenadine	3	502.3	171.1	40	146	40	4	Positive
Citalopram-d6	3	331.44	262.1	50	137	16	4	Positive
Citalopram-d6	3	331.44	109.1	50	137	24	4	Positive

Citalopram	3	325.4	262.1	40	129	16	4	Positive
Citalopram	3	325.4	109.1	40	129	24	4	Positive
Fluconazole	3	307.11	238.1	40	109	12	4	Positive
Fluconazole	3	307.11	220.1	40	109	16	4	Positive
Thiamethoxam-d3	3	295.05	214	50	71	8	4	Positive
Thiamethoxam-d3	3	295.05	131.9	50	71	20	4	Positive
Thiamethoxam	3	292.03	211	40	63	8	4	Positive
Thiamethoxam	3	292.03	181	40	63	20	4	Positive
Venlafaxine-d6	3	284	266.2	50	100	8	4	Positive
Venlafaxine-d6	3	284	64.2	50	100	12	4	Positive
Venlafaxine	3	278	260.2	40	95	8	4	Positive
Venlafaxine	3	278	58.1	40	95	16	4	Positive
Desvenlafaxine	3	264.2	246.2	40	105	8	4	Positive
Desvenlafaxine	3	264.2	58.1	40	105	20	4	Positive
Imidacloprid-d4	3	260.09	213	50	59	12	4	Positive
Imidacloprid-d4	3	260.09	179.1	50	59	16	4	Positive
Imidacloprid	3	256.06	209	40	67	8	4	Positive
Imidacloprid	3	256.06	175.1	40	67	12	4	Positive
Sulfamethoxazole	3	254.06	156	40	94	12	4	Positive
Sulfamethoxazole	3	254.06	92.1	40	94	24	4	Positive
Clothianidin	3	250.02	169.1	40	67	8	4	Positive
Clothianidin	3	250.02	131.9	40	67	12	4	Positive
Bupropion-d9	3	249.12	185	50	92	8	4	Positive
Bupropion-d9	3	249.12	131.1	50	92	28	4	Positive
Methocarbamol	3	242.11	118.1	40	72	8	4	Positive
Methocarbamol	3	242.11	57.1	40	72	25	4	Positive
Bupropion	3	240.12	184	40	86	8	4	Positive
Bupropion	3	240.12	131.1	40	86	24	4	Positive
Lidocaine	3	235.18	86.1	40	100	12	4	Positive
Lidocaine	3	235.18	58.1	40	100	36	4	Positive
Caffeine-c3	3	198.18	140	50	123	16	4	Positive
Caffeine-c3	3	198.18	112.1	50	123	24	4	Positive
4/5-methyl-Benzotriazole	3	134	79.1	40	80	20	4	Positive
4/5-methyl-Benzotriazole	3	134	77.1	40	80	28	4	Positive
1H-Benzotriazole	3	120.06	92	40	75	16	4	Positive
1H-Benzotriazole	3	120.06	65.1	40	75	24	4	Positive
Carbamazepine-d10	4	247	204	50	60	14	4	Positive
Carbamazepine-d10	4	247	201	50	60	16	4	Positive
Carbamazepine	4	237	194.1	40	149	16	4	Positive
Carbamazepine	4	237	179	40	149	36	4	Positive

Table S7 Compound levels of detection (LOD) on the in-house LC/MS/MS were determined after running 7 repeated low level mixed standards, calculating the standard deviation, and multiplying by the t-statistic 3.143. The biota LOD values are an approximation calculated using a standard dry mass of 0.2 g and an extraction value of 2 mL. The dry mass for each biota sample varied slightly. The biota LODs are applicable for spiders, aquatic insects, emergent insects, and fish, as they all were extracted using the same method. The water LODs are also a general approximation to 1000 mL, their total volumes also varied slightly. The initial volume for each water sample was approximately 1000 mL with a final volume of 1 mL.

Compound	Instrument method LOD (ng/mL)	Biota LOD for 0.2 g (ng/g)	1000 mL Water LOD (ng/L)
Imidacloprid	0.72	7.2	0.72
Clothianidin	0.48	4.8	0.48
Thiamethoxam	1.11	11.1	1.11
Bupropion	0.99	9.9	0.99
Carbamazepine	1.06	10.6	1.06
Citalopram	1.45	14.5	1.45
Desvenlafaxine	0.70	7.0	0.70
Fexofenadine	0.64	6.4	0.64
Fluconazole	1.08	10.8	1.08
Guanlyurea	1.11	11.1	1.11
Lidocaine	0.86	8.6	0.86
Metformin	0.87	8.7	0.87
Methocarbamol	2.36	23.6	2.36
Sulfamethoxazole	1.31	13.1	1.31
Venlafaxine	0.71	7.1	0.71
1H-benzotriazole	0.84	8.4	0.84
4/5-methyl-Benzotriazole	0.58	5.8	0.58

SECTION S3: SPIDER Collection, Extraction, Results

Sampling Locations

Three ~70 m transects surrounding the established USGS water sampling sites were created for the spider sampling. At the US1 site, the spider transect was established 20 m upstream and 50 m downstream from the water sampling location. No transect was established near the effluent

discharge pipe because emergent aquatic insects can move both up and downstream. Therefore, we presumed that spiders next to the effluent channel could be consuming insects that spent their larval stage either upstream or downstream from this contaminant point source. The DS1 transect started at the surface water grab sampling location and extended 90 m downstream. The extra 20 m was to make up for a portion of the stream that was inaccessible due to downed trees. The final transect was created at DS2, starting at the surface water grab sampling location under the bridge and extending 70 m downstream.

Spider Collection & Characterization

Spiders from the family Tetragnathidae were collected over a five-day period from August 4, 2020 to August 8, 2020 (**Figure S3**) at the established transects in Muddy Creek. The sampling process occurred at night using the same protocols from Naslund et al., 2020.[8] Spiders were collected with acid washed bone tweezers starting at the stream surface up to 1 meter horizontally and up to 2 meters vertically from the water. The spiders were collected by hand (using tweezers, gloved hands) and each spider was stored in an individual 2 mL propylene vial and then placed on ice. Each night the transect sampling order was changed to avoid any time bias (**Table S8**). This same protocol was also used for a single daytime sampling inside the North Liberty Wastewater Plant on August 10th, 2020, however no spiders were located.

The collected spiders were individually characterized for general size, sex, and identified[9] as tetragnathid upon visual inspection of the eye pattern, front leg length, and chelicera size. The individual spiders were classified as male, female, or juvenile/unknown. The samples were frozen at -20 °C in their individual tubes for further processing of chemical composition.

Table S8 Dates, times, numbers of spiders collected from Muddy Creek in August 2020.

Sample Site	Date	Start Time	Total # Collected	Notes
US1	8/4/2020	9:30 PM	42	1st site, 1 hour (8min per 10m)
DS1	8/4/2020	11:00 PM	43	2nd site, 1 hour (8min per 10m)
DS2	8/4/2020	12:30 AM	35	3rd site, 1 hour (8min per 10m)
DS2	8/5/2020	6:50 PM	31	1st site, 55 min
DS1	8/5/2020	8:10 PM	8	2nd site, 35 min
US1	8/5/2020	9:00 PM	28	3rd site, 45min
DS1	8/6/2020	9:10 PM	41	1st site, 45min
US1	8/6/2020	10:00 PM	20	2nd site, 35 min
DS2	8/6/2020	11:05 PM	31	3rd site, 35 min
DS2	8/8/2020	8:50 PM	63	1st site, 40 min
US1	8/8/2020	9:55 PM	34	2nd site, 40 min
DS1	8/8/2020	10:45 PM	58	3rd site, 40 min

Reference Spider Collection

Spiders were collected from a reference site in North Carolina (NC) that has no known municipal wastewater influence. There are a limited number of septic fields and small farms with livestock, and the watershed is predominately forested. These samples were collected and processed in the same manner as the spiders in Iowa. They were identified as tetragnathid and sexed, placed on ice, and shipped to the lab at the University of Iowa.

Extraction of spider tissues for pharmaceuticals and pesticides

The spiders were stored frozen at -20 °C prior to processing. The individual spiders were freeze-dried on a lyophilizer (Labconco Freezone6) in their original polypropylene tubes. After freeze-drying, the samples were composited based on sex[10] and location (i.e. Upstream Males). After combining the spiders into their identified groups, they were ground and homogenized using a Retsch MM 400 mixer mill. After homogenization, approximately 0.1-0.2 grams of dry tissue was weighed out and mixed with 1 mL of ACN:MeOH (80:20) with 2% formic acid, and 100 µL

surrogate standard mix (500 ng/mL; venlafaxine-d_6, bupropion-d_9, citalopram-d_6, imidacloprid-d_4, carbamazepine-d_{10}). Solvent extraction was completed 1× via bead-beater mixer milling, sonication, and vortexing. Two more extractions were completed with an additional 0.5 mL of solvent each time, for a total of 3 extractions. The 3× supernatant was removed, filtered, and combined in a separate labeled centrifuge tube. Additionally, the combined supernatant was frozen for 24 hours to encourage the separation of residual lipids. After freezing and thawing, the supernatant was again centrifuged again and filtered (0.2 µm, PTFE) into a sample vial, where 10 µL of an internal standard mix (thiamethoxam-d_3, 10 mg/L; caffeine-$^{13}C_3$, 10 mg/L) was added. The samples were stored at -20 °C until analyzed on the LC/MS/MS. See **Section S2** for instrument parameters. Results are listed in **Table S9**.

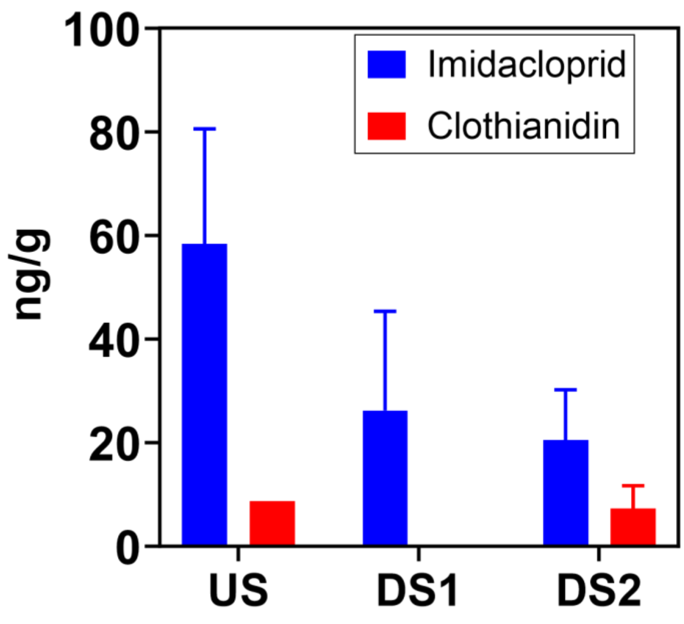

Figure S4 Compiled imidacloprid (blue) and clothianidin (red) concentrations in spider tissues (ng/g) for each site at Muddy Creek. Each bar height represents the average concentration and error bars represent maximum concentration.

Table S9 Numerical results of the target compounds found in the spider tissues collected from Muddy Creek and in the minimally human-impacted reference site in North Carolina (NC). Imidacloprid and clothianidin were detected in the Muddy Creek spiders, but only sulfamethoxazole was detected in the female NC reference spiders. LODs listed in **Table S7.** Spider samples that were unable to be sexed ("other") were not included in calculations due to their low masses.

Sample #	Sex	Site	Dry Mass (g)	Imidacloprid (ng/g)	Clothianidin (ng/g)	Sulfamethoxazole (ng/g)	All other target chemicals
-	Female	NC reference	0.3654	< 7.2	< 4.8	**48**	< LOD
-	Female	NC reference	0.4387	< 7.2	< 4.8	**43**	< LOD
-	Male	NC reference	0.1924	< 7.2	< 4.8	< 13.1	< LOD
-	Male	NC reference	0.1053	< 7.2	< 4.8	< 13.1	< LOD
1	Female	US1	0.237	46.5	< 4.8	< 13.1	< LOD
2	Female	US1	0.1732	45.5	< 4.8	< 13.1	< LOD
3	Male	US1	0.2639	84.0	8.7	< 13.1	< LOD
-	Other	US1	0.0236	< 7.2	< 4.8	< 13.1	< LOD
4	Female	DS1	0.1698	8.9	< 4.8	< 13.1	< LOD
5	Female	DS1	0.1519	11.6	< 4.8	< 13.1	< LOD
6	Male	DS1	0.1579	35.5	< 4.8	< 13.1	< LOD
7	Male	DS1	0.1857	49.2	< 4.8	< 13.1	< LOD
-	Other	DS1	0.0635	< 7.2	< 4.8	< 13.1	< LOD
8	Female	DS2	0.3222	30.0	9.7	< 13.1	< LOD
9	Female	DS2	0.2869	26.8	10.7	< 13.1	< LOD
10	Male	DS2	0.1663	16.0	6.9	< 13.1	< LOD
11	Male	DS2	0.1247	9.1	E 1.2	< 13.1	< LOD
-	Other	DS2	0.0223	< 7.2	< 4.8	< 13.1	< LOD
Mass of all Muddy Creek Spiders			2.3489				
Mass of n=11 Muddy Creek Spiders			2.2395				

Table S10 The ratios of imidacloprid (IMI) to clothianidin (CLO) as fold-changes are listed. Averages with standard deviations are listed and the full ranges of values are listed. For the first 2 columns, water neonic data from Webb et al 2021[5] was used in addition to the August 2020 data for a total n=92. The Iowa Ag site surface water data was pulled from Hladik et al 2014[11] for a total n=18. For August 2020 water samples there was a total of n=16 neonic samples. The spider ratios from August 2020 had a total of n=5 neonic ratios.

Site	Average Water[5] IMI:CLO ratio	Range[5] IMI:CLO ratio	Water IMI:CLO ratio August 2020	Range IMI:CLO ratio August 2020	Spider IMI:CLO ratio August 2020	Range Spiders IMI:CLO ratio August 2020
US1	1.4 ± 1.8	0.04 - 3.7	4.6 ± 0.8	4.0 - 5.9	9.6	N/A
EFF	9.4 ± 21.7	0.13 - 92.3	5.5 ± 1.3	3.7 - 6.9	No spiders collected	No spiders collected
DS1	7.0 ± 15.2	0.16 - 62.5	5.3 ± 1.5	3.8 - 7.5	IMI only in spider	IMI only in spider
DS2	4.6 ± 9.4	0.08 - 44.2	5.7 ± 1.7	3.6 - 7.8	4.0 ± 2.64	2.3 - 7.8
Combined Muddy Creek	5.6 ± 14.0	0.04 - 92.3	5.3 ± 1.3	3.6 - 7.8	5.09 ± 3.42	2.3 - 9.6
Iowa Ag Sites[11]	0.46 ± 0.88	0.03 - 3.95	N/A	N/A	N/A	N/A

Table S11 Surrogate recoveries of deuterated compounds from spider tissues. The surrogate recoveries were not used in calculations – these were for QC purposes only.

Sex	Location	Mass (g)	Venlafaxine-d6	Bupropion-d9	Citalopram-d6	Imidacloprid-d4	Carbamazepine-d10
Female	NC Reference	0.3654	28%	50%	15%	24%	30%
Female	NC Reference	0.4387	26%	49%	13%	20%	33%
Male	NC Reference	0.1924	138%	60%	38%	67%	112%
Male	NC Reference	0.1053	159%	67%	50%	93%	91%
Female	US1	0.2370	98%	57%	27%	57%	67%
Male	US1	0.2639	156%	80%	25%	82%	150%
Other	US1	0.0236	96%	115%	73%	163%	75%
Female	DS1	0.1698	84%	57%	28%	47%	49%
Female	DS1	0.1519	91%	69%	31%	55%	53%
Male	DS1	0.1579	115%	74%	32%	62%	99%
Male	DS1	0.1857	120%	60%	34%	57%	97%
Other	DS1	0.0635	69%	91%	47%	87%	48%
Female	DS2	0.3222	88%	53%	22%	52%	82%
Female	DS2	0.2869	94%	54%	24%	53%	84%
Male	DS2	0.1663	152%	81%	42%	83%	124%
Male	DS2	0.1247	161%	68%	48%	90%	110%
Other	DS2	0.0223	92%	109%	68%	145%	72%
			**Avg.**	**Avg.**	**Avg.**	**Avg.**	**Avg.**
			**104%**	**70%**	**36%**	**73%**	**81%**

SECTION S4: EMERGENT MACROINVERTEBRATE Collection, Extraction, Results

Emergent Insect Collection & Characterization

Emergent insects were collected at the same time as the spiders in August 2020. Emergence traps and sticky traps were deployed for 8 days starting on August 4th, 2020. Along each transect we established 5 floating emergence traps. For each site we tried to put traps (1) over a pool, (2) below/near fallen branches, (3) near the edge of stream bank, (4) near other points of interest (e.g.,

rocks). Sticky emergence traps were also set near each emergence trap on vegetation or overhanding branches in the vicinity (within ~6 ft). Emergent insects from Muddy Creek were collected during the spider collections, but there was insufficient biomass to chemically analyze. Emergent insects from the orders Trichoptera, Ephemeroptera, Odonata, and Diptera were noted – it is likely that tetragnathid spiders prey on these types of insects for food. Unfortunately, the samplers at DS1 were lost and never recovered due to the historic Midwest derecho on August 10th, 2020.

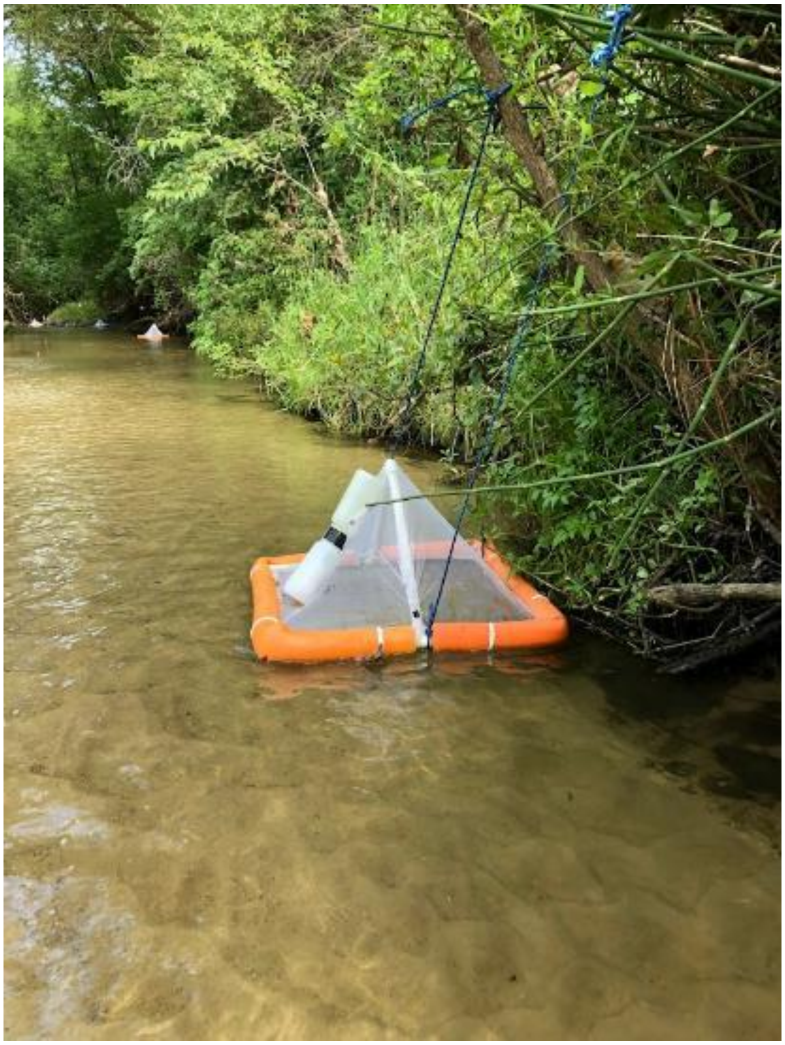

Figure S5 Floating emergence trap in Muddy Creek. Note additional traps in the background. Photo taken by the authors on August 6th, 2020.

Table S12 Emergent insects collected from Muddy Creek on sticky traps and in emergence traps. All the samples from US1 were composited together and all samples except 1 large Odonata were composited from DS2 for the pharmaceutical/pesticide analysis. No compounds were detected in any samples. LOD for all chemicals are listed in **Table S7**.

Site	Bug Mass Wet (mg)	Count	Order	Lab sample	Dry Mass (g)	Chemistry Result
US1	11.9	10	Trichoptera	Composite	0.0253	< LOD
US1	2.2	53	Diptera			
US1	6.4	1	Ephemeroptera			
US1	13.4	4	Trichoptera			
US1	5.7	139	Diptera			
US1	2.3	3	Ephemeroptera			
DS2	11.1	2	Trichoptera	Composite	0.0398	< LOD
DS2	1.9	39	Diptera			
DS2	1.3	21	Diptera			
DS2	40	8	Trichoptera (larger)			
DS2	7.1	4	Trichoptera (smaller)			
DS2	1.1	2	Ephemeroptera			
DS2	241.3	1	Odonata	1x Odonata	0.1887	< LOD

SECTION S5: AQUATIC MACROINVERTEBRATE Collection, Extraction, Results

Macroinvertebrate Collection & Characterization

Aquatic macroinvertebrates were collected from US1, DS1, and DS2 locations in Muddy Creek on July 6th, 2022. Each site was collected for 1 hour using dip nets and kick nets to collect enough biomass for chemical analysis. The captured insects were placed into plastic baggies and placed on ice until further analysis. No aquatic macroinvertebrates were captured at DS2, likely due to the homogeneous sandy conditions. The macroinvertebrates were classified to high level order upon return to the lab. Additionally, concurrent surface water samples were collected at the same time from US, EFF, and DS2 for comparative analysis (**Table S16**, **Figure S6**).

Table S13 Aquatic insects collected from Muddy Creek on July 7th, 2022. A total of 16 organisms were collected from US1 and a total of 52 organisms were collected from the stream space between EFF and DS1. Insects listed in ***bold italics*** correspond to insects that were also found in the emergence traps.

Aquatic Insect	# Collected US1	# Collected EFF/DS1
Ephemeroptera (Mayfly)	***0***	***3***
Trichoptera (Caddisfly)	***4***	***3***
Elmidae (Riffle Beetle)	1	2
Odonata Zygoptera (Damselfly)	***2***	***9***
Odonata Anisoptera (Dragonfly)	***1***	***1***
Amphipod (Scud)	0	1
Gerridae (Water Strider)	5	27
Corixidae (Water Boatman)	3	2
Haliplidae (Crawling Water Beetle)	0	4
Total Count	16	52

Extraction of macroinvertebrate tissues for pharmaceuticals and pesticides

The macroinvertebrates were stored frozen at -20 °C prior to processing. The macroinvertebrates were then separated into 2 groups: US1 insects and DS1 insects. They were then freeze-dried on a lyophilizer (Labconco FreeZone6). After freeze-drying, the grouped samples were ground and homogenized using a Retsch MM 400 mixer mill. After homogenization, approximately 0.1-0.2 grams of dry tissue was weighed out and mixed with 1 mL of ACN:MeOH (80:20) with 2% formic acid, and 100 µL surrogate standard mix (500 ng/mL; venlafaxine-d_6, bupropion-d_9, citalopram-d_6, imidacloprid-d_4, carbamazepine-d_{10}). Solvent extraction was completed 1× via bead-beater mixer milling, sonication, and vortexing. Two more extractions were completed with an additional 0.5 mL each time, for a total of 3 extractions. The 3× supernatant was removed, filtered, and combined in a separate labeled centrifuge tube. Additionally, the combined supernatant was frozen for 24 hours to encourage the separation of residual lipids. After freezing and thawing, the

supernatant was again centrifuged again and filtered (0.2 μm, PTFE) into a sample vial, where 10 μL of an internal standard mix (thiamethoxam-d_3, 10 mg/L and caffeine-$^{13}C_3$, 10 mg/L) was added. The samples were stored at -20 °C until analyzed on the LC/MS/MS. See **Section S2** for instrument parameters.

Table S14 Pharmaceuticals detected in the aquatic insects collected in July 2022. US1 insects listed in **Table S13** were composited into a single sample and aquatic insects collected from DS1 were composited into 3 samples. LODs are listed in **Table S7**.

Site	Dry Mass (g)	Bupropion (ng/g)	Citalopram (ng/g)	Fexofenadine (ng/g)	All other chemicals
**US1**	0.0664	< 9.9	< 14.5	< 6.4	< LOD
**DS1**	0.1368	51	19	6.7	< LOD
**DS1**	0.1256	< 9.9	17	6.7	< LOD
**DS1**	0.1733	< 9.9	16	E 5.9	< LOD

SECTION S6: FISH Collection, Extraction, Results

Fish Collection & Characterization

Fish were collected via an electrofishing survey from the US1, DS1, and DS2 locations in Muddy Creek on July 19th, 2019. Approximately 100 meter transects were shocked using backpack electro-fishing units, and stunned fish were netted by hand. Starting downstream, the samplers slowly moved upstream collecting fish until reaching the end location. All fish were placed into coolers and identified to species. Rare fish species (*e.g.,* Johnny Darter) were returned to the creek unharmed. The sampled fish were individually frozen until further analysis. Additionally, concurrent surface water samples were collected at the same time from US, EFF, DS1, and DS2 for comparative analysis.

Extraction of fish tissues for pharmaceuticals and pesticides

The fish were stored frozen at -20 °C prior to processing. They were then individually freeze-dried on a lyophilizer (Labconco FreeZone6). After freeze-drying, the individual samples were ground and homogenized using a Retsch MM 400 mixer mill. After homogenization, approximately 0.1-0.2 grams of dry tissue was weighed out and mixed with 1 mL of ACN:MeOH (80:20) with 2% formic acid, and 100 µL surrogate standard mix (500 ng/mL; venlafaxine-6, bupropion-d9, citalopram-d6, imidacloprid-d4, carbamazepine-d10). Solvent extraction was completed 1x via bead-beater mixer milling, sonication, and vortexing. Two more extractions were completed with an additional 0.5 mL each time, for a total of 3 extractions. The 3x supernatant was removed, filtered, and combined in a separate labeled centrifuge tube. Additionally, the combined supernatant was frozen for 24 hours to encourage the separation of residual lipids. After freezing and thawing, the supernatant was again centrifuged again and filtered (0.2 µm, PTFE) into a sample vial, where 10 µL of an internal standard mix (thiamethoxam-d^3, 10 mg/L and caffeine-$^{13}C_3$, 10 mg/L) was added. The samples were stored at -20 C° until analyzed on the LC/MS/MS. See **Section S2** for instrument parameters. Results in **Table S15**.

Table S15 Pharmaceutical results for fish collected from Muddy Creek in July 2019. Some results listed as estimated (E) because they were less than the original calculated instrument LOD, but obvious peaks were observed. LODs are listed in **Table S7**.

Site	Species	Dry Mass (g)	Bupropion (ng/g)	Lidocaine (ng/g)	Venlafaxine (ng/g)	Citalopram (ng/g)	All other chemicals
US1	Shiner	0.4138	< 9.9	< 8.6	< 7.1	< 14.5	< LOD
US1	Shiner	0.4236	< 9.9	< 8.6	< 7.1	< 14.5	< LOD
US1	Shiner	0.423	< 9.9	< 8.6	< 7.1	< 14.5	< LOD
US1	Chub	0.4106	< 9.9	< 8.6	< 7.1	< 14.5	< LOD
US1	Chub	0.4114	< 9.9	< 8.6	< 7.1	< 14.5	< LOD
US1	Chub	0.3996	< 9.9	< 8.6	< 7.1	< 14.5	< LOD
US1	Chub	0.413	< 9.9	< 8.6	< 7.1	< 14.5	< LOD
US1	Chub	0.4086	< 9.9	< 8.6	< 7.1	< 14.5	< LOD
US1	Chub	0.415	< 9.9	< 8.6	< 7.1	< 14.5	< LOD
DS1	Bluntnose Minnow	0.4174	E 2.2	< 8.6	E 5.44	< 14.5	< LOD
DS1	Bluntnose Minnow	0.4025	E 1.24	< 8.6	E 2.67	< 14.5	< LOD
DS1	Bluntnose Minnow	0.4144	E 1.07	< 8.6	E 2.55	< 14.5	< LOD
DS1	Chub	0.3985	E 1.14	< 8.6	E 2.72	E 3.04	< LOD
DS1	Chub	0.4141	< 9.9	< 8.6	< 7.1	E 3.39	< LOD
DS1	Chub	0.4184	E 1.13	< 8.6	E 3.18	E 3.03	< LOD
DS1	Chub	0.4084	E 1.78	< 8.6	E 2.71	E 2.04	< LOD
DS1	Chub	0.4247	E 1.65	< 8.6	E 2.89	E 2.02	< LOD
DS1	Chub	0.4099	E 1.59	< 8.6	E 2.73	E 2.24	< LOD
DS2	Fathead Minnow	0.4648	E 2.23	10.9	E 4.04	< 14.5	< LOD
DS2	Fathead Minnow	0.3983	E 1.45	11.2	E 4.21	< 14.5	< LOD
DS2	Shiner	0.4228	E 1.91	< 8.6	E 2.35	< 14.5	< LOD
DS2	Shiner	0.4188	E 1.08	< 8.6	< 7.1	< 14.5	< LOD
DS2	Shiner	0.4108	< 9.9	< 8.6	< 7.1	< 14.5	< LOD
DS2	Bigmouth Shiner	0.4285	E 2.61	< 8.6	7.11	< 14.5	< LOD
DS2	Bigmouth Shiner	0.4047	E 2.61	< 8.6	7.21	< 14.5	< LOD
DS2	Bigmouth Shiner	0.4253	E 2.51	< 8.6	E 6.88	E 2.37	< LOD

SECTION S7: SURFACE WATER Collection, Extraction, Results

Water Sample Collection - USGS

Water samples were collected from the 4 established sites during the morning of August 6, 2020 by a USGS researcher. The samples were immediately filtered, placed on ice, and shipped to the USGS National Water Quality Laboratory in Denver, Colorado. The samples were analyzed for 110 human use pharmaceuticals via direct injection analysis[3] =Results are in **Table S17** and data release.[2]

Grab Sample Collection - UIowa

Water samples were collected from the 4 established sites on August 3, 6, 13, in 2020 to fully capture the spider sampling campaign. The samples were always collected in the same order (US1, EFF, DS1, DS2) and 2 x 1L samples were collected during each sampling instance. Acid washed 1L glass amber bottles were used to collect the grab samples, using the vertical centroid-of-flow method.[3] After collection, samples were immediately placed on ice until they arrived back in the lab, which took no more than 4 hours. The samples were vacuum filtered (Whatman GF/F, nominal 0.7 μm) within 8 hours of collection. Samples were frozen at -20 °C in their original sample bottles until extraction, which occurred within 3 months. Results are shown in **Figure S6** with neonicotinoid results for clothianidin, imidacloprid, and thiamethoxam listed in **Table S16.**

Solid Phase Extraction of water for pharmaceuticals and pesticides

<u>Waters HLB extraction - neonicotinoids</u>

Water samples were first measured volumetrically, then fortified with 100 μL of a surrogate mixture (50 ng imidacloprid-d_4). SPE was performed manually using a Visiprep SPE manifold

with vacuum pump. First, cartridges were gravity conditioned with 5 mL dichloromethane, followed by 5 mL acetone, followed by 10 mL optima water. Samples were loaded onto cartridges with vacuum assistance at a maximum speed of 10 mL/min. After sample loading, the cartridges were dried under vacuum for 30 minutes. Each cartridge was gravity eluted with 10 mL of acetone/dichloromethane (50/50, v/v). After elution, each sample was evaporated to dryness under a gentle stream of nitrogen using an Organomation N-EVAP 11. The dry residues were reconstituted with 1 mL of acetonitrile/optima water (50/50, v/v) and filtered through a 0.2 μm PTFE syringe filter into a glass amber HPLC vial. Finally, 10 μL of an internal standard (thiamethoxam-d^3, 10 mg/L) was added to each sample. If the samples were not analyzed by LC/MS/MS immediately, they were stored at -20 C° and then analyzed within 14 days. See **Section S2** for instrument parameters.

UIowa Water Results

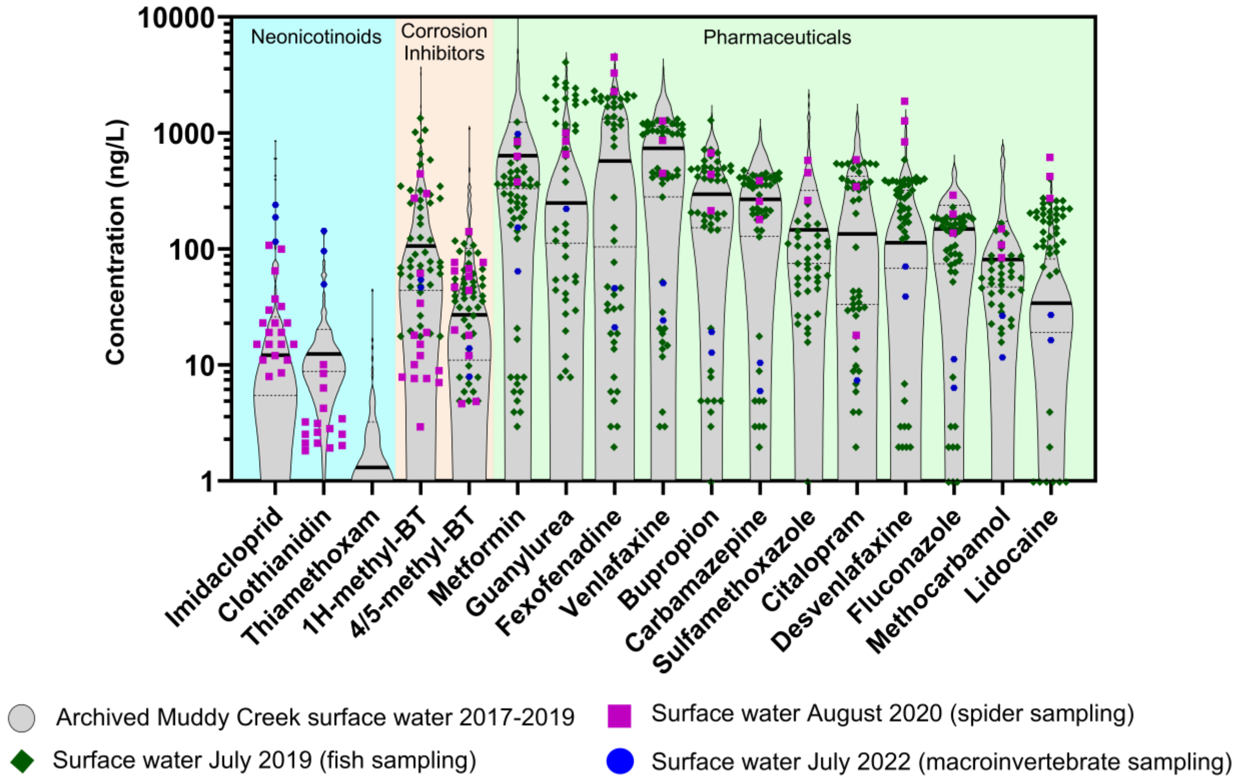

Figure S6 Contaminants in Muddy Creek are continuously released from the North Liberty Wastewater Treatment Plant. Archived water samples from Muddy Creek were collected biweekly by University of Iowa researchers[4,5,12] from 2017-2019 (grey shaded). Upper and lower quartiles are denoted by dashed lines and median values by a solid line. Water samples from July 2019[4,12] (green diamonds ◆) were collected by UIowa 3x a day for 6 days and coincide with collected fish samples. Water samples from August 2020 (purple squares ■) were collected 1x a day for 3 days by UIowa (neonicotinoids) and USGS (pharmaceuticals) and coincide with collected spider samples. Water samples from July 2022 (blue circles ●) were collected 1x on 1 day by UIowa and coincide with collected aquatic insect samples.

Table S16 Surface water neonicotinoid numerical results from Muddy Creek in ng/L in Muddy Creek water as measured from the UIowa laboratory during spider collections and during aquatic insect collections. The average surrogate recovery of imidacloprid-d4 was 78.5% (range: 58.9% - 107.5%; standard deviation: 10.2%).

UIowa neonicotinoid SW August 2020 (spider)				
**Location**	**Date**	**Clothianidin**	**Imidacloprid**	**Thiamethoxam**
Extraction Blank	8/3/2020	< 0.48	< 0.72	< 1.11
US1	8/3/2020	2.5	10.6	< 1.11
EFF	8/3/2020	10.0	36.6	< 1.11
DS1	8/3/2020	8.40	31.8	< 1.11
DS2	8/3/2020	6.29	22.9	< 1.11
US1	8/6/2020	1.77	7.89	< 1.11
US1 duplicate	8/6/2020	2.10	8.45	< 1.11
EFF	8/6/2020	4.15	23.3	< 1.11
EFF duplicate	8/6/2020	3.23	19.2	< 1.11
DS1	8/6/2020	2.76	14.9	< 1.11
DS1 duplicate	8/6/2020	3.09	14.7	3.4
DS2	8/6/2020	2.56	14.9	< 1.11
DS2 duplicate	8/6/2020	2.12	12.4	< 1.11
US1	8/13/2020	1.86	11.1	< 1.11
EFF	8/13/2020	3.39	23.2	< 1.11
DS1	8/13/2020	2.55	18.9	< 1.11
DS2	8/13/2020	1.97	15.4	< 1.11
UIowa neonicotinoid SW July 2022 (aquatic insect)				
Extraction Blank	7/6/2022	< 0.48	< 0.72	< 1.11
US1	7/6/2022	145	244	< 1.11
DS1	7/6/2022	97	190	< 1.11
DS2	7/6/2022	50	117	< 1.11

US Geological Survey Water Results

Table S17 U.S. Geological Survey (USGS) pharmaceutical and pesticide results in ng/L from Muddy Creek collected on August 6th, 2020. These samples were processed at the National Water Quality Laboratory located in Denver, CO. Bold italic pharmaceuticals represent the 12 compounds represented in the UIowa method and in **Figure S6**. Data are available from the USGS National Water Information System database.[2]

USGS Measured Compound (ng/L)	US1 (05454050)	EFF (05454051)	DS1 (05454052)	DS2 (05454090)
1H-1,2,4-Triazole	<56.6	<87.7	<89.1	<79.9
2,4-D	37.5	<62.0	63.3	34.5
2-Chloro-4-isopropylamino-6-amino-s-triazine	37.4	10.1	17.2	21.4
2-Chloro-6-ethylamino-4-amino-s-triazine	14.4	<20.0	<20.0	<20.0
2-Hydroxy-4-isopropylamino-6-amino-s-triazine	E7.71	<16.0	<4.00	<4.00
2-Hydroxy-4-isopropylamino-6-ethylamino-s-triazine	E113	<22.1	E47.8	E85.8
3-Phenoxybenzoic acid	<200	<200	<200	<200
4-Hydroxychlorothalonil	<100	<100	<100	E511
Acephate	<10.0	<10.0	<10.0	<10.0
Acetochlor oxanilic acid	E50.8	<100	<100	E22.1
Acetochlor sulfonic acid	<400	<400	<400	<400
Acetochlor	<25.0	<25.0	<25.0	<25.0
Atrazine	E69.5	12.5	26.4	37.9
Azoxystrobin	E15.6	3.75	6.62	11.7
Bentazon	<9.00	<9.00	<9.00	<9.00
Bromacil	<10.0	<10.0	<10.0	<10.0
Bromoxynil	<60.0	<60.0	<60.0	<60.0
Carbaryl	<5.60	<5.60	<5.60	<5.60
Carbendazim	E6.73	E26.0	E20.9	E32.3
Chlorimuron-ethyl	<25.0	<25.0	<25.0	<25.0
Chlorodiamino-s-triazine	E65.0	E75.5	E64.5	E55.6
Chlorpyrifos	<3.00	<3.00	<3.00	<3.00
cis-Cyhalothric acid	<500	<500	<500	<500
cis-Permethrin	<4.2	<4.2	<4.2	<4.2
Dechlorometolachlor	E29.4	<2.00	7.82	7.23
Desulfinylfipronil	<10.0	1.27	<10.0	1.42
Diazinon	<6.40	<6.40	<6.40	<6.40

Dicamba	<800	<800	<800	<800
Dichlorvos	<52.0	<52.0	<52.0	<52.0
Dicrotophos	<10.0	<10.0	<10.0	<10.0
Diflubenzuron	<6.00	<6.00	<6.00	<6.00
Diketonitrile-isoxaflutole	<25.0	<25.0	<25.0	<25.0
Dimethenamid	E4.23	<3.00	<3.00	4.62
Dimethoate	<4.60	<4.60	<4.60	<4.60
Diuron	<5.00	17	14.4	21.9
Ethoprop	<5.00	<5.00	<5.00	<5.00
Etoxazole	<4.20	<4.20	<4.20	<4.20
Fipronil amide	<9.20	10.9	6.88	5.34
Fipronil sulfide	<4.20	E3.61	E2.42	E1.99
Fipronil sulfone	<5.60	9.18	6.12	4.54
Fipronil	<4.00	46.2	33.7	19.4
Fluometuron	<10.0	<10.0	<10.0	<10.0
Halosulfuron methyl	<12.0	<12.0	<12.0	<12.0
Hexazinone	<3.60	<3.60	<3.60	<3.60
Hydroxymetolachlor	44.1	<5.00	9.75	7.08
Hydroxysimazine	<120	<120	<120	<120
Imazethapyr	<10.0	<10.0	<10.0	<10.0
Imidacloprid	29.7	108	65.1	100
Linuron	<5.60	<5.60	<5.60	<5.60
Malathion	<10.0	<10.0	<10.0	<10.0
Metalaxyl	<6.00	<6.00	<6.00	<6.00
Metconazole	1.89	<5.00	<5.00	6.11
Methamidophos	<25.0	<25.0	<25.0	<25.0
Methomyl	<3.00	<3.00	<3.00	<3.00
Methoxyfenozide	<2.20	1.07	<2.20	0.86
Metolachlor oxanilic acid	E306	<149	E131	E122
Metolachlor sulfonic acid	646	342	444	365
Metolachlor	E208	E3.5	E47.0	E32.7
Metribuzin	<20.0	<20.0	<20.0	<20.0
Myclobutanil	<7.00	<7.00	<7.00	<7.00
N-(3,4-Dichlorophenyl)-N'-methylurea	E1.70	<5.00	<5.00	8.56
Oryzalin	<50.0	<50.0	<50.0	<50.0
Piperonyl butoxide	<25.0	<25.0	<25.0	<25.0
Prometon	3.14	2.3	2.51	6.61
Propazine	<3.20	<3.20	<3.20	<3.20
Propiconazole	13.1	4.76	5.76	9.68
Propoxur	<3.20	<3.20	<3.20	<3.20
Pyraclostrobin	E1.49	<2.40	1.28	1.68

Simazine	<10.0	<10.0	<10.0	<10.0
Sulfentrazone	<50.0	15.9	11.9	6.73
Sulfometuron-methyl	<10.0	<10.0	<10.0	<10.0
Tebuconazole	<15.0	65.2	48.2	89.7
Tebupirimfos	<2.00	<2.00	<2.00	<2.00
Tebuthiuron	6.13	1.5	2.84	<3.00
Terbufos sulfone	<50.0	<50.0	<50.0	<50.0
Terbufos sulfoxide	<3.00	<3.00	<3.00	<3.00
Terbufos	<6.80	<6.80	<6.80	<6.80
Tetraconazole	<7.00	<7.00	<7.00	<7.00
Thiabendazole	<4.00	E17.9	E13.1	E6.58
Thiobencarb	<4.20	<4.20	<4.20	<4.20
trans-Permethrin	<3.80	<3.80	<3.80	<3.80
Triclopyr	<88.0	<88.0	<88.0	<88.0
Trifloxystrobin	<2.80	<2.80	<2.80	6.64
1,7-Dimethylxanthine	<88.0	<88.0	<88.0	<88.0
10-Hydroxy-amitriptyline	<8.30	E31.9	E21.2	E7.91
Abacavir	<4.00	<4.00	<4.00	<4.00
Acetaminophen	<20.0	<22.0	<20.0	<22.4
Acyclovir	<80.0	E2000	E1430	E634
Albuterol	<6.70	E11.2	E7.97	E4.80
Alprazolam	<21.0	4.02	<21.0	<21.0
Amitriptyline	<37.0	<37.0	E9.44	<37.0
Amphetamine	<4.40	<4.40	<4.40	<4.40
Antipyrine	<50.0	<50.0	<50.0	<50.0
Atenolol	<13.0	E67.5	E55.8	E29.2
Benztropine	<44.0	<44.0	<44.0	<44.0
Betamethasone	<114	<114	<114	<114
Bupropion	***<18.0***	***683***	***444***	***216***
Caffeine	<91.0	<91.0	<91.0	E27.9
Carbamazepine	***<20.0***	***392***	***260***	***181***
Carisoprodol	<20.0	<20.0	<20.0	<20.0
Chlorpheniramine	<54.0	E26.9	E22.2	<54.0
Cimetidine	<140	E296	E240	E143
Citalopram	***<6.60***	***595***	***348***	***18***
Clonidine	<61.0	<61.0	<61.0	<61.0
Codeine	<32.0	53.2	38.7	<32.0
Cotinine	<6.40	11.1	10.2	7.97
Dehydronifedipine	<20.0	<20.0	<20.0	<20.0
Desmethyldiltiazem	<70.0	E14.3	<70.0	<70.0
Desvenlafaxine	***<84.0***	***E1910***	***E1290***	***E851***

Dextromethorphan	<8.20	37.5	24.1	10.4
Diazepam	<4.00	<4.00	<4.00	<4.00
Diltiazem	<20.0	E49.2	E34.2	E13.8
Diphenhydramine	<48.0	181	108	<48.0
Duloxetine	<37.0	15.4	<37.0	<37.0
Erythromycin	<80.0	<80.0	<80.0	<80.0
Ezetimibe	<205	<205	<205	<205
Fadrozole	<13.0	<13.0	<13.0	<13.0
Famotidine	<34.0	E469	E337	E166
Fenofibrate	<20.0	<20.0	<20.0	<20.0
Fexofenadine	***<44.0***	***E4610***	***E3350***	***E2320***
Fluconazole	***<30.0***	***E294***	***E201***	***E138***
Fluoxetine	<26.0	62.9	42.7	<26.0
Fluticasone propionate	<30.0	<30.0	<30.0	<30.0
Fluvoxamine	<80.0	<80.0	<80.0	<80.0
Gabapentin	<160	661	468	264
Glipizide	<80.0	<80.0	<80.0	<80.0
Glyburide	<58.0	<58.0	<58.0	<58.0
Guanlyurea	***<140***	***1020***	***864***	***662***
Hexamethylenetetramine	<110	E159	E121	E116
Hydrocodone	<40.0	<40.0	<40.0	<40.0
Hydrocortisone	<147	<147	<147	<147
Hydroxyzine	<20.0	4.36	<20.0	<20.0
Iminostilbene	<200	<200	<200	<200
Ketoconazole	<113	<113	<113	<113
Lamivudine	<16.0	<16.0	<16.0	<16.0
Lidocaine	***<8.00***	***623***	***427***	***275***
Loperamide	<80.0	<80.0	<80.0	<80.0
Loratadine	<7.00	E5.76	E4.63	E2.59
Lorazepam	<202	<202	<202	<202
Meprobamate	<12.0	7.61	<12.0	5.21
Metaxalone	<20.0	<20.0	<20.0	<20.0
Metformin	***<20.0***	***E853***	***638***	***383***
Methadone	<7.60	3.57	3.16	<7.60
Methocarbamol	***<11.0***	***150***	***109***	***84.3***
Methotrexate	<52.0	<52.0	<52.0	<52.0
Methyl-1H-benzotriazole	<80.0	E449	E303	E276
Metoprolol	<10.0	339	219	131
Morphine	<80.0	<80.0	<80.0	<80.0
Nadolol	<20.0	<20.0	<20.0	<20.0
Nevirapine	<46.0	<46.0	<46.0	<46.0

Nicotine	<58.0	<58.0	<58.0	<58.0
Nizatidine	<80.0	<80.0	<80.0	<80.0
Nordiazepam	<20.0	<20.0	<20.0	<20.0
Norethindrone	<80.0	<80.0	<80.0	<80.0
Norfluoxetine	<80.0	<80.0	<80.0	<80.0
Norsertraline	<80.0	<80.0	<80.0	<80.0
Norverapamil	<10.0	E7.60	<10.0	<10.0
Omeprazole + Esomeprazole	<16.0	E10.1	<16.0	<16.0
Oseltamivir	<20.0	<20.0	<20.0	<20.0
Oxazepam	<226	<226	<226	<226
Oxycodone	<25.0	<25.0	<25.0	<25.0
Paroxetine	<72.0	<72.0	<72.0	<72.0
Penciclovir	<80.0	<80.0	<80.0	<80.0
Pentoxifylline	<9.40	<9.40	<9.40	<9.40
Phenazopyridine	<20.0	<20.0	<20.0	<20.0
Phendimetrazine	<20.0	<20.0	<20.0	<20.0
Phenytoin	<400	<400	<400	<400
Piperonyl butoxide	<80.0	E0.81	<80.0	<80.0
Prednisolone	<150	<150	<150	<150
Prednisone	<105	<105	<105	<105
Promethazine	<200	<200	<200	<200
Propoxyphene	<28.0	<28.0	<28.0	<28.0
Propranolol	<26.0	145	99.9	40.6
Pseudoephedrine + Ephedrine	<6.00	9.46	6.55	9.71
Quinine	<80.0	E29.8	E25.3	E15.2
Ractopamine	<20.0	<20.0	<20.0	<20.0
Raloxifene	<80.0	<80.0	<80.0	<80.0
Ranitidine	<192	E225	E120	E56.4
Sertraline	<16.0	E106	E51.6	<16.0
Sitagliptin	<97.0	163	124	54
Sulfadimethoxine	<30.0	<30.0	<30.0	<30.0
Sulfamethizole	<104	<104	<104	<104
Sulfamethoxazole	***<20.0***	***588***	***460***	***264***
Tamoxifen	<270	<270	<270	<270
Temazepam	<20.0	31.5	<20.0	<20.0
Theophylline	<200	<200	<200	<200
Tiotropium	<50.0	<50.0	<50.0	<50.0
Tramadol	<7.40	290	193	119
Triamterene	<5.20	103	71.8	36.2
Trimethoprim	<20.0	45.2	29.5	8.02
Valacyclovir	<163	<163	<163	<163

Venlafaxine	***<5.20***	***1290***	***875***	***455***
Verapamil	<140	E18.1	E13.1	<140
Warfarin	<20.0	6.16	6.02	<20.0

Table S-18 Surrogate recoveries for U.S. Geological Survey pharmaceutical method. Data available from the USGS National Water Information System database.[2]

USGS Surrogate (% Recovery)	US1 (05454050)	EFF (05454051)	DS1 (05454052)	DS2 (05454090)
2,4-D-d3	93.7	93.1	96.5	88.4
3-Phenoxybenzoic acid-13C6	98.4	101	101	102
Acetaminophen-d3	51	41.4	37.6	43.2
Acetochlor-d11	113	94.7	96.3	108
Alachlor-d13	99.4	108	101	98.5
Albuterol-d9	78	78.8	74.7	84
Amitriptyline-d3	113	125	117	94.7
Amphetamine-d6	104	93.8	89.4	99.1
Butachlor sulfonic acid	82.6	82.2	83.8	79.3
Caffeine-(trimethyl-13C3)	104	101	88.9	104
Carbaryl-d7	101	109	94.7	56.2
Carbendazim-d4	94.9	100	96.5	97.1
Carbofuran-d3 (N-methyl-d3)	102	107	104	95.4
Carisoprodol-d7	109	99.5	93	100
cis-Permethrin-13C6	58	100	69.8	80.2
Codeine-d6	94.6	81.9	80.3	89.4
Cotinine-d3	102	99.6	93.2	101
Deethylatrazine-d6	98.8	91.4	90.3	87.4
Diazepam-d5	101	97.1	92.9	103
Diazinon-d10 (diethyl-d10)	116	109	103	111
Diflubenzuron-d4 (-chlorophenyl-d4)	101	112	107	106
Diltiazem-d3	84.5	99.5	84	82.5
Dimethachlor sulfonic acid	86.5	86.2	90.3	90.8
Diphenhydramine-d3	118	114	109	119
Diuron-d6	98.6	111	103	100
Erythromycin-13C-d3	66.8	101	75.3	89.5
Ezetimibe-d4	30	53.6	37.5	16.1
Fenofibrate-d6	65.5	79.7	58.1	49
Fexofenadine-d10	102	104	98.5	93.2
Fluoxetine-d6	98	104	83.4	57.2
Fluvoxamine-d4	97.9	100	81.1	73
Hexazinone-d6 (N	96.5	107	103	101

Hydrocodone-d3	112	99	105	106
Hydrocortisone-2	98.1	96.1	86.3	76.3
Ketoconazole-d4	78.9	47	29.1	16.9
Linuron-d6 (dimethyl-d6)	99.6	114	103	104
Loperamide-d6	86.3	94.6	79.2	46
Loratadine-d4	89.7	92.2	69	42.1
Lorazepam-d4	113	131	118	126
Malathion-d10 (diethyl-d10)	90.5	102	88.5	41.2
Metformin-d6	90.8	52.2	53.1	64.5
Methadone-d9	122	117	107	104
Metolachlor-d6	100	112	101	104
N-Desmethyldiltiazem-d4	98.2	105	97.1	86.1
Nicosulfuron-d6 (dimethoxy-d6)	107	115	104	108
Norfluoxetine-d6	91.5	83.5	87.5	84.6
Oxazepam-d5	85.3	102	109	128
Oxycodone-d3	103	103	93.3	107
Promethazine-d6	108	52.7	46.5	42.9
Propoxyphene-d11	113	113	102	105
Pseudoephedrine-d3	99	95	87.5	97.3
Raloxifene-d10	39.9	61.7	57.9	26.9
Ranitidine-d6	30.6	25.5	38.9	53.7
Sulfamethoxazole-(phenyl-13C6)	98.3	98.4	90.7	107
Tamoxifen-d5	E38.5	E101	E116	E61.0
Tebuconazole-d6	104	119	110	102
Temazepam-d5	111	107	102	111
Thiabendazole-d4	110	101	99.5	111
Thiobencarb-d10 (diethyl-d10)	106	115	106	108
Tiotropium-d3	32.7	90.1	78.8	20.3
Trimethoprim-d9	96.3	91.1	88.1	104
Verapamil-d6	66.3	108	82.1	67

SECTION S8: REFERENCES

(1) United States Census Bureau. Quick Facts: North Liberty city, Iowa https://www.census.gov/quickfacts/northlibertycityiowa (accessed Jan 17, 2024).

(2) US Geological Survey. USGS 05454050 Muddy Cr abv Trtmnt Outfall at North Liberty, IA https://waterdata.usgs.gov/nwis/inventory/?site_no=05454050&agency_cd=USGS&amp (accessed May 14, 2024). https://doi.org/10.5066/F7P55KJN.

(3) Furlong, E. T.; Noriega, M. C.; Kanagy, C. J.; Kanagy, L. K.; Coffey, L. J.; Burkhardt, M. R. Determination of Human-Use Pharmaceuticals in Filtered Water by Direct Aqueous Injection—High-Performance Liquid Chromatography/Tandem Mass Spectrometry: U.S. Geological Survey Techniques and Methods. In *Methods of the National Water Quality Laboratory*; Reston, VA, 2014; p 49. https://doi.org/10.3133/tm5B10.

(4) Zhi, H.; Kolpin, D. W.; Klaper, R. D.; Iwanowicz, L. R.; Meppelink, S. M.; Lefevre, G. H. Occurrence and Spatiotemporal Dynamics of Pharmaceuticals in a Temperate-Region Wastewater Effluent-Dominated Stream: Variable Inputs and Differential Attenuation Yield Evolving Complex Exposure Mixtures. *Environ. Sci. Technol.* **2020**, *54* (20), 12967–12978. https://doi.org/10.1021/acs.est.0c02328.

(5) Webb, D. T.; Zhi, H.; Kolpin, D. W.; Klaper, R. D.; Iwanowicz, L. R.; LeFevre, G. H. Emerging Investigator Series: Municipal Wastewater as a Year-Round Point Source of Neonicotinoid Insecticides That Persist in an Effluent-Dominated Stream. *Environ. Sci. Process. Impacts* **2021**, *23* (5), 678–688. https://doi.org/10.1039/D1EM00065A.

(6) LeFevre, G. H.; Müller, C. E.; Li, R. J.; Luthy, R. G.; Sattely, E. S. Rapid Phytotransformation of Benzotriazole Generates Synthetic Tryptophan and Auxin Analogs in Arabidopsis. *Environ. Sci. Technol.* **2015**, *49* (18), 10959–10968. https://doi.org/10.1021/acs.est.5b02749.

(7) Shoda, M. E.; Nowell, L. H.; Stone, W. W.; Sandstrom, M. W.; Bexfield, L. M. Data Analysis Considerations for Pesticides Determined by National Water Quality Laboratory Schedule 2437. *U.S. Geol. Surv. Sci. Investig. Rep.* **2018**. https://doi.org/10.3133/sir20185007.

(8) Naslund, L. C.; Gerson, J. R.; Brooks, A. C.; Walters, D. M.; Bernhardt, E. S. Contaminant Subsidies to Riparian Food Webs in Appalachian Streams Impacted by Mountaintop Removal Coal Mining. *Environ. Sci. Technol.* **2020**, *54* (7), 3951–3959. https://doi.org/10.1021/acs.est.9b05907.

(9) Bradley, R. A. *Common Spiders of North America*, 1st ed.; University of California Press, 2012.

(10) Beaubien, G. B.; Olson, C. I.; Otter, R. R. The Role of Sexual Dimorphism and Tissue Selection in Ecotoxicological Studies Using the Riparian Spider Tetragnatha Elongata. *Bull. Environ. Contam. Toxicol.* **2019**, *103* (2), 225–232. https://doi.org/10.1007/s00128-019-02632-y.

(11) Hladik, M. L.; Kolpin, D. W.; Kuivila, K. M. Widespread Occurrence of Neonicotinoid Insecticides in Streams in a High Corn and Soybean Producing Region, USA. *Environ. Pollut.* **2014**, *193*, 189–196. https://doi.org/10.1016/j.envpol.2014.06.033.

(12) Meppelink, S. M.; Kolpin, D. W.; Lane, R. F.; Iwanowicz, L. R.; Zhi, H.; Lefevre, G. H. *Water-Quality Data for a Pharmaceutical Study at Muddy Creek in North Liberty and Coralville, Iowa, 2017-2018: U.S. Geological Survey Data Release*; 2020. https://doi.org/10.5066/P9WOD2XB.

(13) U.S. Environmental Protection Agency. Comptox Chemicals Dashboard v2.2.1.

(14) Williams, A. J.; Grulke, C. M.; Edwards, J.; McEachran, A. D.; Mansouri, K.; Baker, N. C.; Patlewicz, G.; Shah, I.; Wambaugh, J. F.; Judson, R. S.; Richard, A. M. The CompTox Chemistry Dashboard: A Community Data Resource for Environmental Chemistry. *J. Cheminform.* **2017**, *9* (1), 61. https://doi.org/10.1186/s13321-017-0247-6.

(15) Mansouri, K.; Grulke, C. M.; Judson, R. S.; Williams, A. J. OPERA: A Free and Open Source QSAR Tool for Predicting Physicochemical Properties and Environmental Fate Endpoints. New Orleans, LA 2018.

(16) Martin, T. M. User's Guide for T. E. S. T. (Toxicity Estimation Software Tool) Version 5.1. 2020.

(17) U.S. Environmental Protection Agency. *Bioaccumulation Testing and Interpretation for the Purpose of Sediment Quality Assessment, Status and Needs.*; Washington, D.C., 2000.

(18) U.S. Environmental Protection Agency. *TSCA New Chemicals Program (NCP) Chemical Categories*; Washington, D.C., 2010.

(19) Roodt, A. P.; Huszarik, M.; Entling, M. H.; Schulz, R. Aquatic-Terrestrial Transfer of Neonicotinoid Insecticides in Riparian Food Webs. *J. Hazard. Mater.* **2023**, *455*, 131635. https://doi.org/10.1016/J.JHAZMAT.2023.131635.

(20) Previšić, A.; Vilenica, M.; Vučković, N.; Petrović, M.; Rožman, M. Aquatic Insects Transfer Pharmaceuticals and Endocrine Disruptors from Aquatic to Terrestrial Ecosystems. *Environ. Sci. Technol.* **2021**, *55* (6), 3736–3746. https://doi.org/10.1021/acs.est.0c07609.

(21) Richmond, E. K.; Rosi, E. J.; Walters, D. M.; Fick, J.; Hamilton, S. K.; Brodin, T.; Sundelin, A.; Grace, M. R. A Diverse Suite of Pharmaceuticals Contaminates Stream and Riparian Food Webs. *Nat. Commun.* **2018**, *9* (1), 4491. https://doi.org/10.1038/s41467-018-06822-w.
